# Novel Google Maps and Google Earth application for chemical industry disaster risk assessment during complex emergencies in Eastern Ukraine

**DOI:** 10.1038/s41598-023-31848-6

**Published:** 2023-04-08

**Authors:** Rick Kye Gan, Emanuele Bruni, Rafael Castro Delgado, Carlos Alsua, Pedro Arcos González

**Affiliations:** 1 Unit for Research in Emergency and Disaster, Public Health Area, Department of Medicine, University of Oviedo, Oviedo, Asturias Spain; 2 Department of Global Public Health, Karolinska Institutet, Stockholm, Sweden; 3 Servicio de Salud del Principado de Asturias (SAMU-Asturias), Instituto de Investigación Sanitaria del Principado de Asturias, Oviedo, Asturias Spain; 4 McGuire Center for Entrepreneurship, University of Arizona, Tucson, USA

**Keywords:** Environmental impact, Public health

## Abstract

The war in Ukraine has led to complex emergencies, humanitarian crises, and other severe consequences, such as chemical industry disasters. The chemical industry is one of the principal sectors of Ukraine’s economy. In 2019, Ukraine had a total volume of hazardous chemical accumulation of more than a 5.1billion tons. Therefore, an attack on chemical industrial facilities will lead to catastrophic consequences such as chemical disasters. This paper aims to study the disaster risk of chemical industrial facilities and its effects on public health and the environment during complex emergencies in Eastern Ukraine. Observational cross-sectional risk assessment method was utilized to assess hazard, vulnerability, and exposure of the chemical industry in Eastern Ukraine in Donetsk Oblast and Luhansk Oblast. Data on chemical factories in Eastern Ukraine was collected on Google Maps and Google Earth on May 2022. Lastly, the semi-quantitative risk assessment method was utilized to describe the risk from the perspective of consequences for life and health, the environment, property, and speed of development. Our disaster risk assessment found more than 1 million people (1,187,240 people) in Donetsk Oblast and more than 350 thousand people (353,716 people) in Luhansk Oblast are exposed to potential hazards from the chemical facilities clusters. The aggregation risk of bombardment of chemical facilities cluster in Eastern Ukraine is also high due to ongoing war. Therefore, the chemical industry disaster risks for Eastern Ukraine during complex emergencies in Donetsk Oblast and Luhansk Oblast are high in terms of likelihood and consequences to life and health, environment, property, and speed of development.

## Introduction

Since mid-May 2014, armed conflicts in Eastern Ukraine have been fought along a contact line separating government-controlled areas from non-governmental-controlled areas in Donetsk Oblast and Luhansk Oblast^1^. The conflict escalated into a war on the 24th of February 2022 when the Russian military entered Ukraine^1,2^. The war in Ukraine has led to complex emergencies and humanitarian crises and other severe consequences, such as chemical industry disasters.

Industrial production is an essential characteristic of the modern world economy. Nevertheless, alongside economic development comes the risk of industrial hazards that affect public health and the environment. Examples of hazards are toxic releases, explosions, fires, and chemical spills. Examples of hazards are toxic releases, explosions, fires, and chemical spills^3^. The chemical industry is one of the principal sectors of Ukraine’s economy. In 2019, the Ukrainian chemical industry output reached 2.8 billion USD^4^. On the other hand, it is estimated that Ukraine has a total volume of hazardous chemical accumulation of more than a 5.1billion tons^5^. In early March 2022, the European Union expressed deep concern via OPCW (Organisation for the Prohibition of Chemical Weapons) about the information that the Russian military forces are possibly preparing ‘false flag’ provocations in Ukraine using chemicals, including blowing up industrial tanks with chemicals^6^.

An attack on chemical industrial facilities and their storage could lead to chemical incidents. “Chemical incident” can be defined as any uncontrolled release of a toxic substance, potentially harming public health and the environment. A chemical incident can occur due to natural or technological disasters^7^. In Ukraine, industrial sites where hazardous chemicals are produced, stored, or transported could be possible sources of toxic exposure, especially if attacked or damaged by indiscriminate shelling^7^. Unfortunately, the storage or manufacturing facilities that contain toxic industrial chemicals and other toxic materials are located near urban centers^8^. Therefore, an attack on chemical facilities will be the functional equivalent of a chemical weapon attack^8^.

Since the war in Ukraine, the World Health Organization has reported several chemical incidents as a direct result of the armed conflict^9,10^. For example, on the 21st of March 2022, the World Health Organization reported an ammonia leak at an industrial site close to Sumy. In addition, leakage of nitric acid was reported on the 5th of April in Rubizhne in the Luhansk oblast^10–12^. However, no disaster risk analysis has been published for the chemical industry during the complex emergencies in Ukraine. Disaster risk assessment during complex emergencies is essential for the community to understand and communicate potential risks that could bring significant consequences to public health and the environment. However, unlike during normal conditions, disaster risk assessment during complex emergencies is limited by sensitive information and a lack of data.

We hypothesize that the chemical industry disaster risk in Eastern Ukraine is high due to the density of chemical factories in the region and the high aggregation risk of bombardment due to ongoing complex emergencies. Therefore, the chemical industry disaster risk in Eastern Ukraine is high in terms of likelihood and consequences to life and health, environment, property, and speed of development.

## Aim

This paper aimed to assess the disaster risk of chemical industrial facilities and its potential consequences to public health and the environment during complex emergencies in Eastern Ukraine, where limited data is available.

## Method

**Figure 1:**
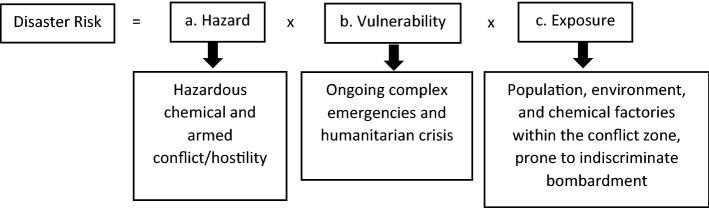
Conceptual framework for this disaster risk assessment adapted from UNDRR terminology and Crichet et al^14,15^.

The study design of this disaster risk assessment utilized an observational cross-sectional risk assessment method. Data on the chemical industries in Eastern Ukraine, such as company name, translated name, type of chemical factory, address, GPS coordination, and Google plus code, were extracted from Google Maps and Google Earth on May 2022. The risk assessment described in this paper applied the conceptual framework adapted from UNDRR terminology and Crichton et al., whereby *disaster risk* is defined as the potential loss of life, injury, or destroyed or damaged assets that could occur to a system, society, or a community in a specific period of time^13^. Therefore, disaster risk is dependent on three elements, (a) hazard, (b) vulnerability, and (c) exposure, as shown in Fig. 1.

### Hazard

A *hazard* is defined as a process, phenomenon, or human activity that may cause loss of life, injury or other health impacts, property damage, social and economic disruption, or environmental degradation^16^. This risk assessment focused on hazardous chemicals from the chemical industry facilities and armed conflict. For instance, there will be higher risk in a location with a higher number of chemical facilities and ongoing armed conflict or hostility.

### Vulnerability

*Vulnerability* is defined as the conditions determined by physical, social, economic, and environmental factors or processes which increase the susceptibility of an individual, a community, assets, or systems to the impacts of hazards^17^. It is well established that complex emergencies and humanitarian crises will increase the vulnerabilities of all levels of the population^18^. Therefore, in this risk assessment, locations in Ukraine with ongoing armed conflict or hostility that gave rise to complex emergencies and humanitarian crises are considered highly vulnerable.

### Exposure

*Exposure* is defined as the situation of people, infrastructure, housing, production capacities, and other tangible human assets located in hazard-prone areas^19^. In this risk assessment, the population of Eastern Ukraine of Donetsk Oblast and Luhansk Oblast, property, environment, and the chemical industrial facilities within the conflict zone were considered high susceptibility.

Lastly, we utilized the semi-quantitative method to further describe the risk from the perspective of consequences for life and health, the environment, property, and speed of development. Semi-quantitative methods are helpful in this risk assessment because the ongoing war in Ukraine affected the access to data collection in Eastern Ukraine. Semi-quantitative methods can be a stepping stone toward complete quantitative research. Nevertheless, meanwhile enable capturing subjective opinion, opening debate, and forming a framework to identify where additional analytical efforts are needed.

### Methodology

In order to address hazard, vulnerability, and exposure, this risk assessment utilizes the following steps:

- **Step 1**: Identify the location of chemical industrial facilities in Eastern Ukraine in Donetsk Oblast and Luhansk Oblast.
- **Step 2**: Identify the cluster of chemical industrial facilities.
- **Step 3**: Determine the type of chemical factory and the most probable chemical hazard.
- **Step 4:** Determine the number of populations exposed to the potential chemical hazard.
- **Step 5**: Based on information gathered from all previous steps, plot a disaster risk metric with the likelihood of the chemical industry being bombarded and consequences addressing life and health, environment, cost of property damage, and speed of development.

All steps are described in detail below:

**Step 1: Identify the location of the chemical industrial facility**

A chemical industrial facility is defined as any structure(s) used or intended for use as a business enterprise for manufacturing, processing, or assembling chemical products or commodities^20^.

Using Google Maps, we identified the chemical industrial facility in Donetsk Oblast and Luhansk Oblast. The search strategy utilized keywords such as ''chemical factory'', ''chemical plant'', and ''chemical industry'' in Donetsk Oblast and Luhansk Oblast. We recorded data such as company name, translated name, type of chemical factory, address, GPS coordination, and Google plus code.

**Figure 2:**
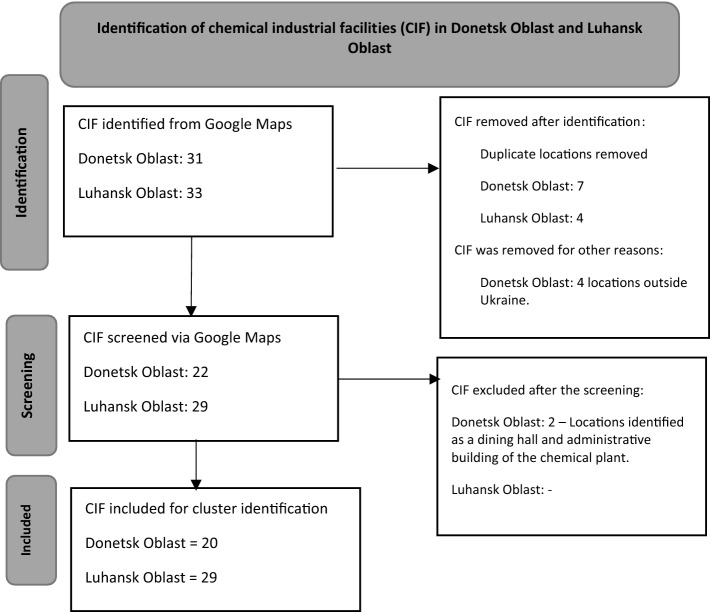
Process of chemical industrial facility identification in Eastern Ukraine.

Google Maps was chosen as the tool to locate chemical industrial facilities because no other best available database could provide the location, type of factories, and GPS coordination in Eastern Ukraine. The process is illustrated in Fig. 2.

**Step 2: Identify the chemical facilities cluster**

**Figure 3:**
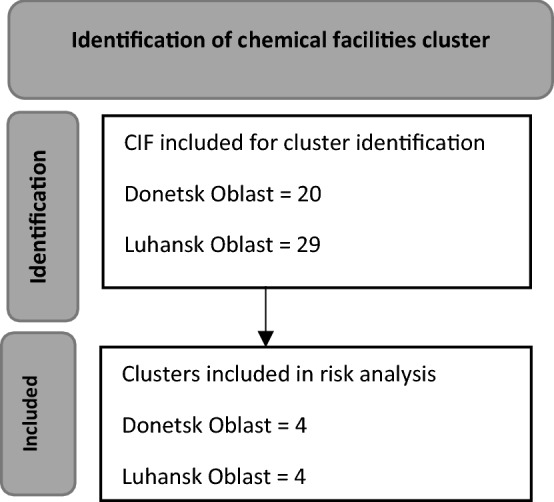
Process of chemical facilities cluster identification in Eastern Ukraine.

The identified chemical industrial facilities were then plotted in Google Earth Pro. Clusters of chemical facilities were identified, as shown in Fig. 3. We defined a chemical facilities cluster as two or more chemical factories within a 5 km radius. Chemical facilities clusters represent the density of chemical industrial facilities in a given location and are associated with compound risk.

**Step 3:** **Identify the hazardous chemicals from the chemical facilities cluster**

The type of chemical facilities based on their manufactured product were identified within all chemical facilities clusters. Owing to the limited information available regarding the type and amount of chemicals used as precursors and produced by the chemical factories in Eastern Ukraine, an assumption on the type of chemical hazard has to be made based on the type of activity and manufacturing production of the factories.

The chemical facilities cluster included registered chemical factories with unknown manufactured products. The reason for including a registered chemical factory with unknown manufactured products is that although there is no available information about the type of chemicals manufactured in these factories, their presence within the chemical facilities cluster reflects the density of chemical factories. The higher the density of chemical industrial facilities, the higher the likelihood of it being bombarded by the ongoing armed conflict, leading to compounded risk and more severe consequences.

**Figure 4:**
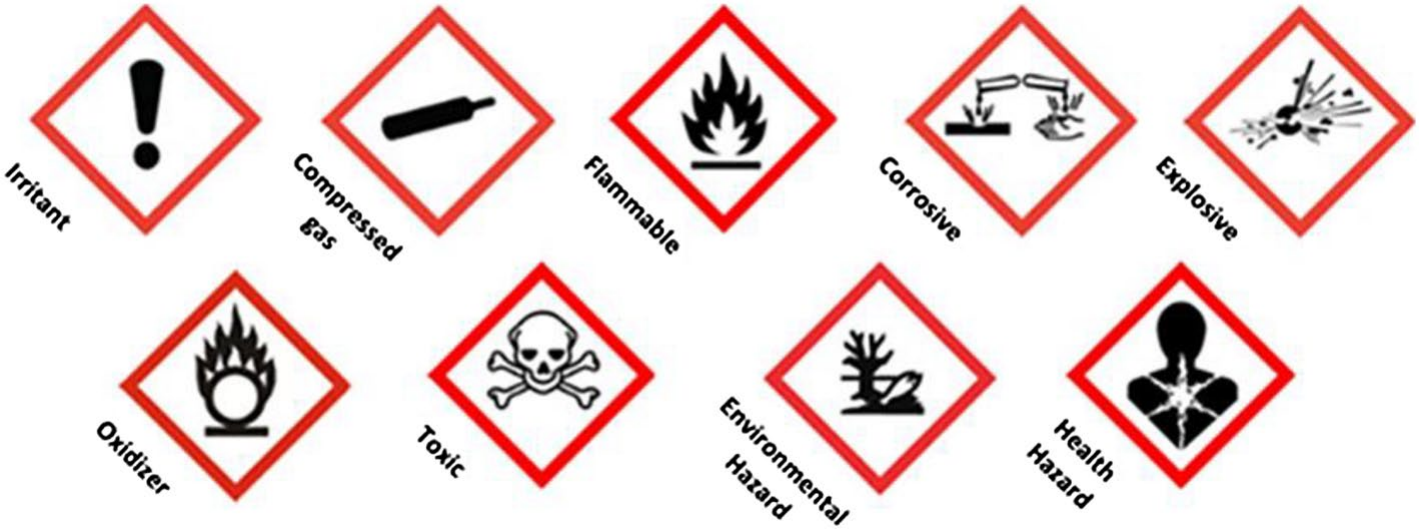
GHS pictograms.

The probable classification based on the Globally Harmonized System of Classification and Labelling of Chemicals (GHS)^21^ was given to every chemical factory based on their type of activity and manufactured product. GHS classification is illustrated in Fig. 4. This step will enable us to identify and classify the hazard.

However, since the exact detail of the type and amount of chemicals used as precursors and produced by the factories could not be determined, assumptions on the type of chemical hazard must be made based on the type of activity and manufactured product of the factories. A detailed analysis of the type of potential chemical hazard can be found in Annex 1.

**Figure 5:**
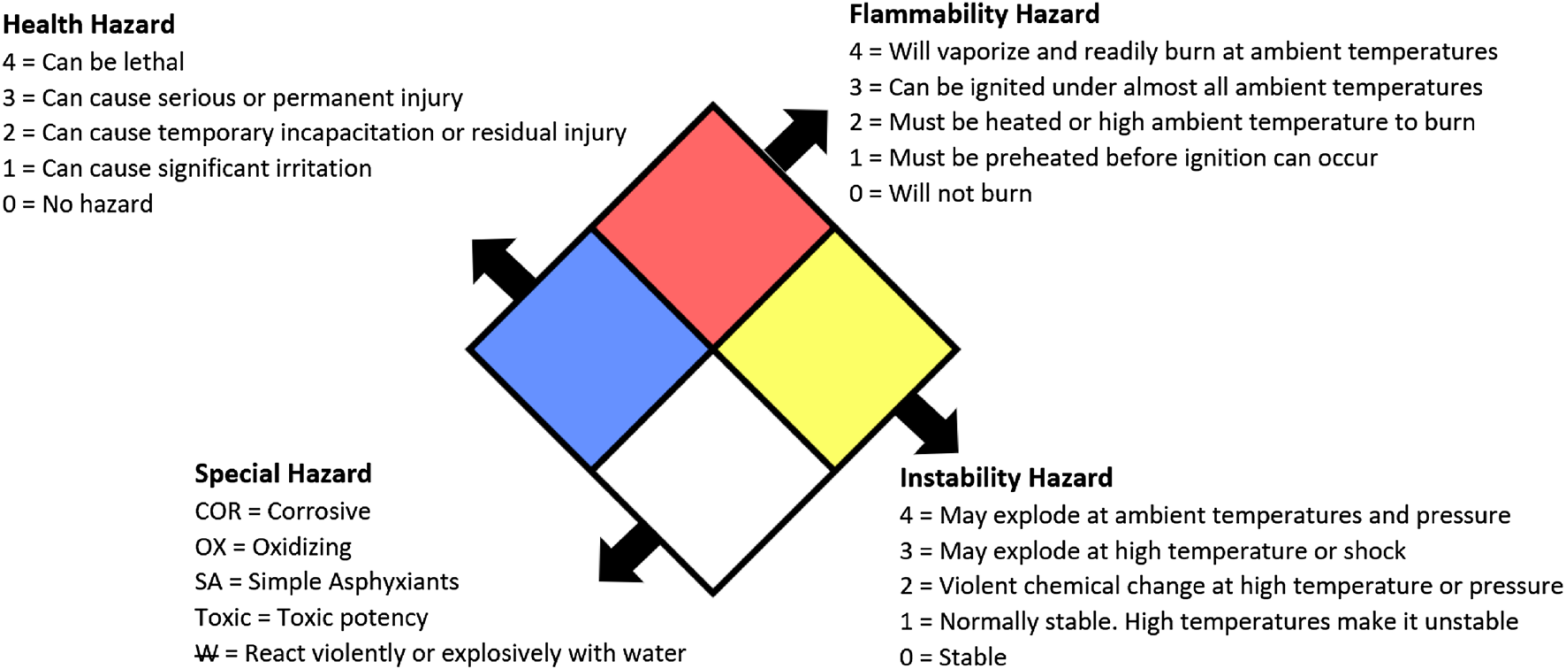
NFPA rating explanation guide.

Potential hazards of every chemical factory were cumulatively described using NFPA 704 (Standard System for the Identification of the Hazards of Materials for Emergency Response)^22^. NFPA 704 has four main headings, namely health hazard (blue/left), flammability hazard (red/top), instability hazard (yellow/right), and special hazard (white/bottom), as shown in Fig. 5.

A final NFPA Hazard Diamond was plotted for all the chemical facilities clusters by listing the most significant hazard from each heading. NFPA Hazard Diamond will provide simple, readily recognized, and easily understood system marking for all the clusters, which provide an immediate general sense of the severity of these hazards and relate to emergency response^22^.

**Step 4:** **Determine the number of populations exposed to the potential chemical hazard**

The city where the chemical facilities cluster is located was noted, and the neighboring city and settlement were identified. Finally, the number of populations residing in the identified city and settlement was determined using the Ukrainian 2022 population census^23^.

**Step 5: Plot the risk matrix**

Risk matrices were plotted with the x-axis representing the consequences of the chemical factory being bombarded and the y-axis representing the likelihood of the chemical factory being bombarded. The score was given by expert judgment from all authors.

We estimated the consequences by considering the character of the hazard, exposure, and vulnerability of the involved location.

The consequence of a chemical factory being bombarded is described under the following indicator:

- Consequences for life and health
- Consequences for the environment
- Consequences for property (total cost of damage in Million USD)
- Speed of development

The details of each indicator, as described in Table 1, were scored based on the scale adapted from APELL’s (Awareness and Preparedness for Emergencies at Local Level) Hazard Identification and Evaluation in a Local Community^24^. Finally, the average score was plotted in the risk matrix.

**Table 1:** Indicator of the consequence of a chemical factory being bombarded.

a. Consequence for life and health	b. Consequence for the environment	c. Consequences for property (total cost of damage Million USD)	d. Speed of development
1. Unimportant—temporary discomfort 2. Limited—A few injuries, long-lasting discomfort 3. Serious—A few serious injuries, serious discomfort 4. Very serious—A few (more than 5) death, several (20) serious injuries, and up to 500 evacuated 5. Catastrophic—Several deaths (more than 20), hundreds of serious injuries, and more than 500 evacuated	1. Unimportant—No contamination, localised effects 2. Limited—Simple contamination, localised effects 3. Serious—Simple contamination, widespread effects 4. Very serious—Heavy contamination, localised effects 5. Catastrophic—Very heavy contamination, widespread effects	1. Unimportant—< 0.5 2. Limited—0.5—1 3. Serious—1—5 4. Very serious—5—20 5. Catastrophic—> 20	1. Early and clear warning—Localised effect/no damage 2. – 3. Medium—Some spreading/small damage 4. – 5. No warning—Hidden until the effects are fully developed/immediate effect(explosion)

We determined the likelihood of a chemical factory being bombarded by classifying the Oblasts into the followings:

- Ongoing armed conflict/hostility: high likelihood
- Resolved armed conflict/hostility: moderate likelihood
- No armed conflict/hostility: low likelihood

## Results

### Donetsk Oblast

**Figure 6:**
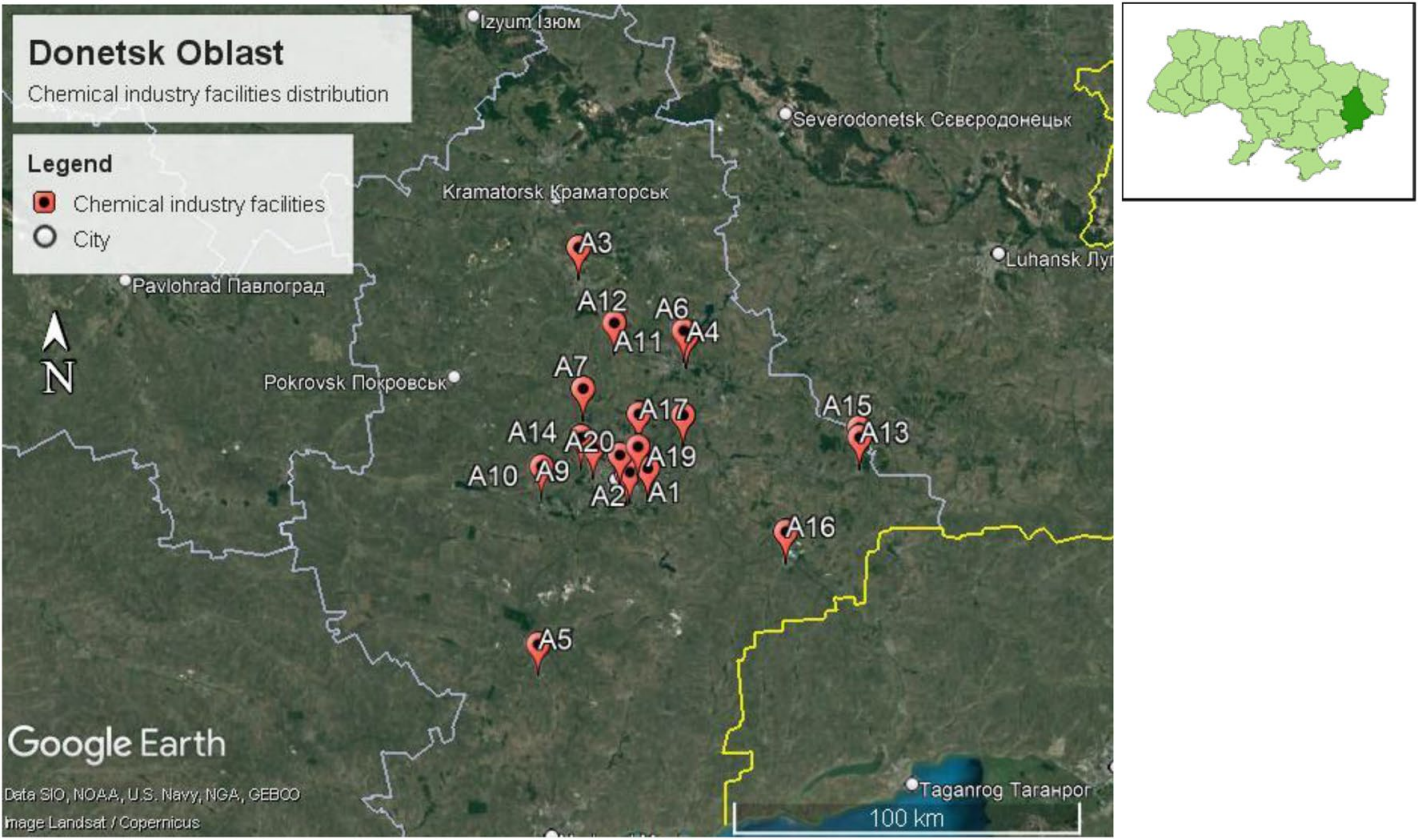
Chemical plant distribution in Donetsk Oblast in Ukraine.

**Figure 7:**
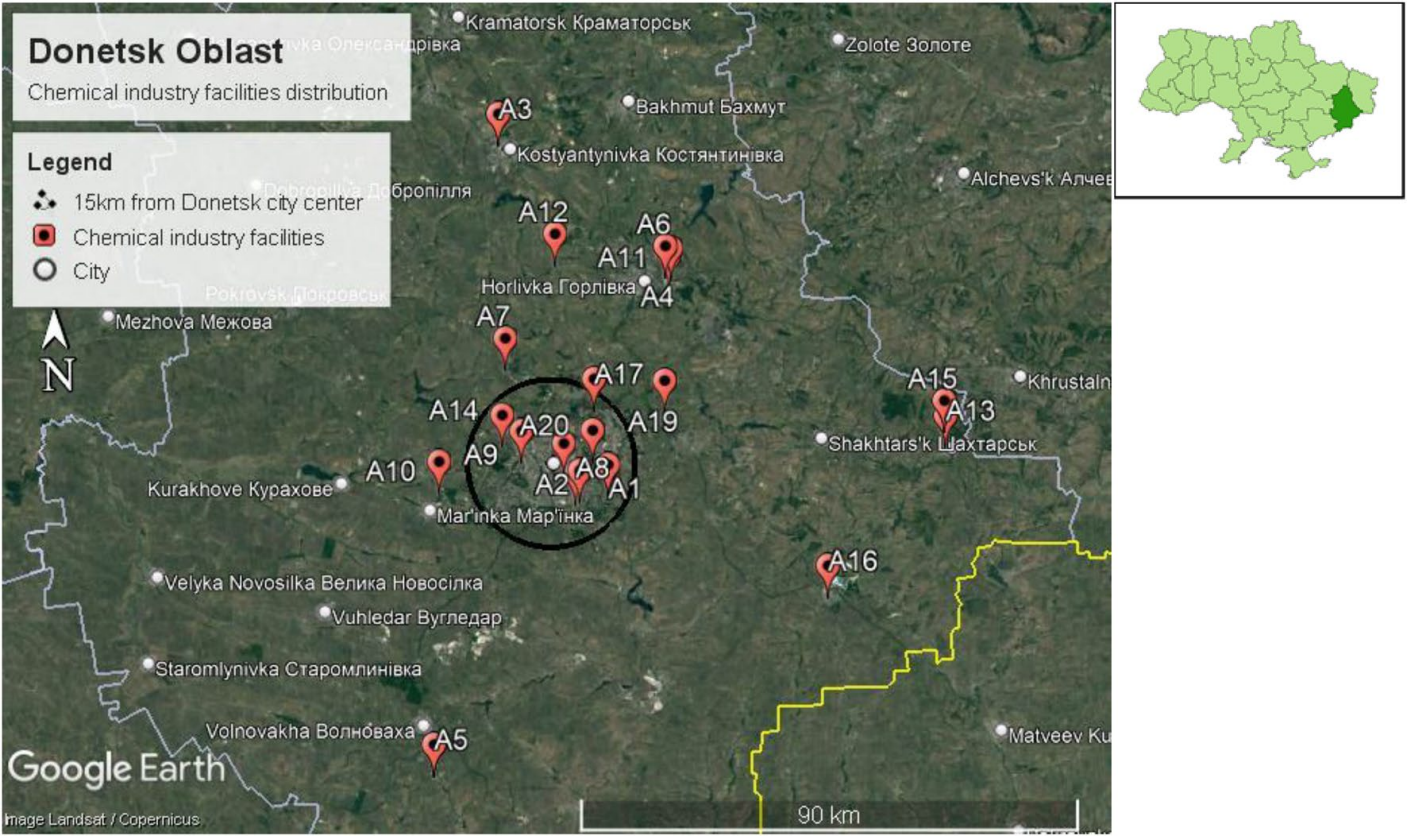
Chemical factories identified within 15 km radius from Donetsk city centre.

The capital of Donetsk Oblast is Donetsk city, located between 48.0159° N, 37.8028° E . The size of Donetsk city is 26,517 km^2^, with a population of 4,100,280, according to the Ukrainian 2022 population census estimation. Therefore, the population density of Donetsk Oblast is 154.6 persons/km^2^. Twenty chemical factories were identified in Donetsk Oblast, as shown in Fig. 6. Moreover, seven chemical factories were identified within a 15 km radius of Donetsk city center, as shown in Fig. 7.

**Figure 8:**
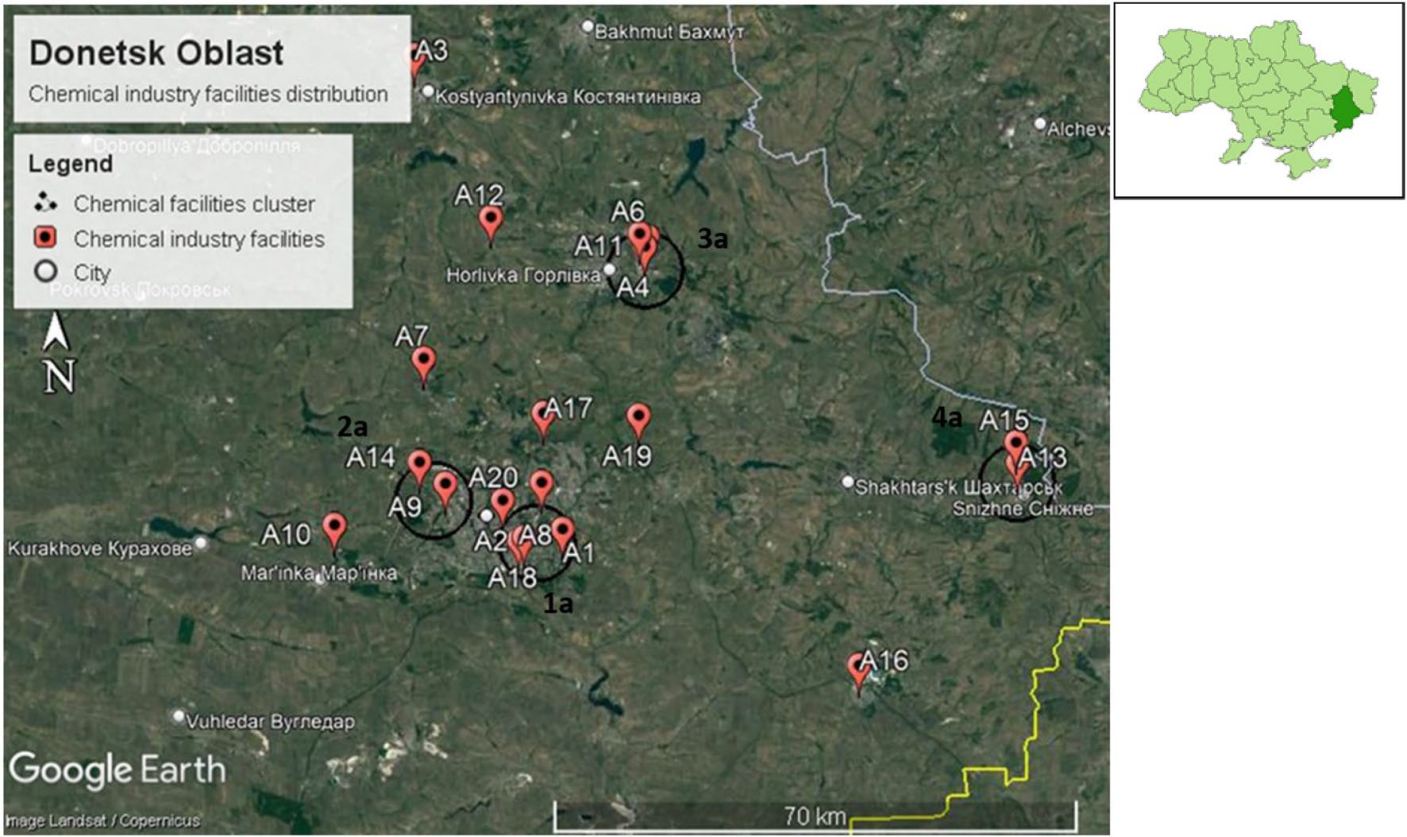
Four chemical facilities cluster identified in Donetsk Oblast.

We identified four chemical facility clusters in Donetsk Oblast, namely clusters 1a, 2a, 3a, and 4a, as shown in Fig. 8. Among the location of the chemical facilities cluster, two clusters are located within Donetsk city (clusters 1a and 2a), 1 cluster within Horlivka city (cluster 3a), and 1 cluster within Snizhne city (cluster 4a).

**Cluster 1a** is located in Donetsk city, with an estimated population of 901,645.

Neighboring cities and settlements for cluster 1a are Makiivka, Yasynuvata, Nyzhnia, Khartsyzk, Proletarske, Zuhres, Shakhtne, Ilovaisk, Mospyne, Pavlohradske, Marianvka, Andriivka, Kirovskyi, Petrovskyi, Staromykhailivka, Lozove, Pisky, and Vesele. These cities and settlements are located in the Donetskyi district, with an estimated population of 1,484,514.

**Cluster 2a** is also located in Donetsk city, with an estimated population of 901,645 persons.

Neighboring cities and settlements for cluster 2a are Makiivka, Yasynuvata, Nyzhnia, Khartsyzk, Proletarske, Zuhres, Shakhtne, Ilovaisk, Mospyne, Pavlohradske, Marianvka, Andriivka, Kirovskyi, Petrovskyi, Staromykhailivka, Lozove, Pisky, and Vesele. These cities and settlements are located in the Donetskyi district, with an estimated population of 1,484,514.

**Cluster 3a** is located in Horlivka city, with an estimated population of 239,828.

Neighboring cities and settlements for cluster 3a are Kalynivka, Vuhlehirsk, Kayutyne, Sofiivka, Novoselivka, Fedorivka, Ozeryanivka, Shyroka Balka, Zalizne, Shumy, Zaitseve, Holmivskyi, and Lozove. These cities and settlements are located in the Horlivskyi district, with an estimated population of 662,069.

**Cluster 4a** is located in Snizhne city, with an estimated population of 45,767 persons.

Neighboring cities and settlements for cluster 4a are Zalisne, Pervomaiske, Pervomaiskyi, Chystiakove, Sukhivske, Siewerne, and Andriivka. These cities and settlements are located in the Horlivskyi district, with an estimated population of 662,069 .

**Figure 9:**
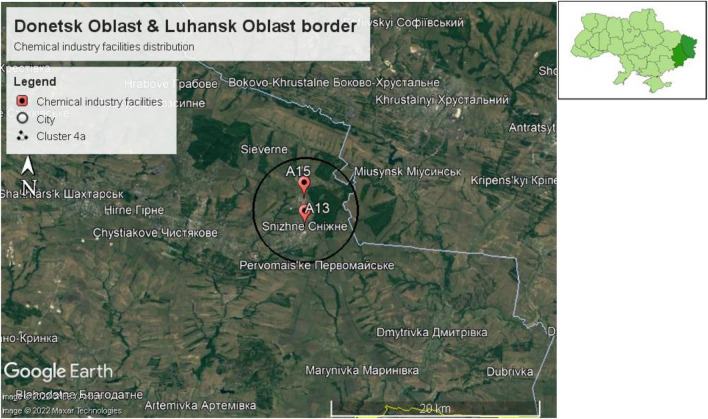
Cluster 4a located near the border of Luhansk Oblast.

**Cluster 4a** is also located on the border of Luhansk Oblast, as shown in Fig. 9. Neighboring cities and settlements for **cluster 4a** in Luhansk Oblast are Bokovo-Khrustalne, Knyahnivka, and Miusynsk. These cities and settlements are located in the Rovenkivskyi district, with an estimated population of 294,125.

**Figure 10:**
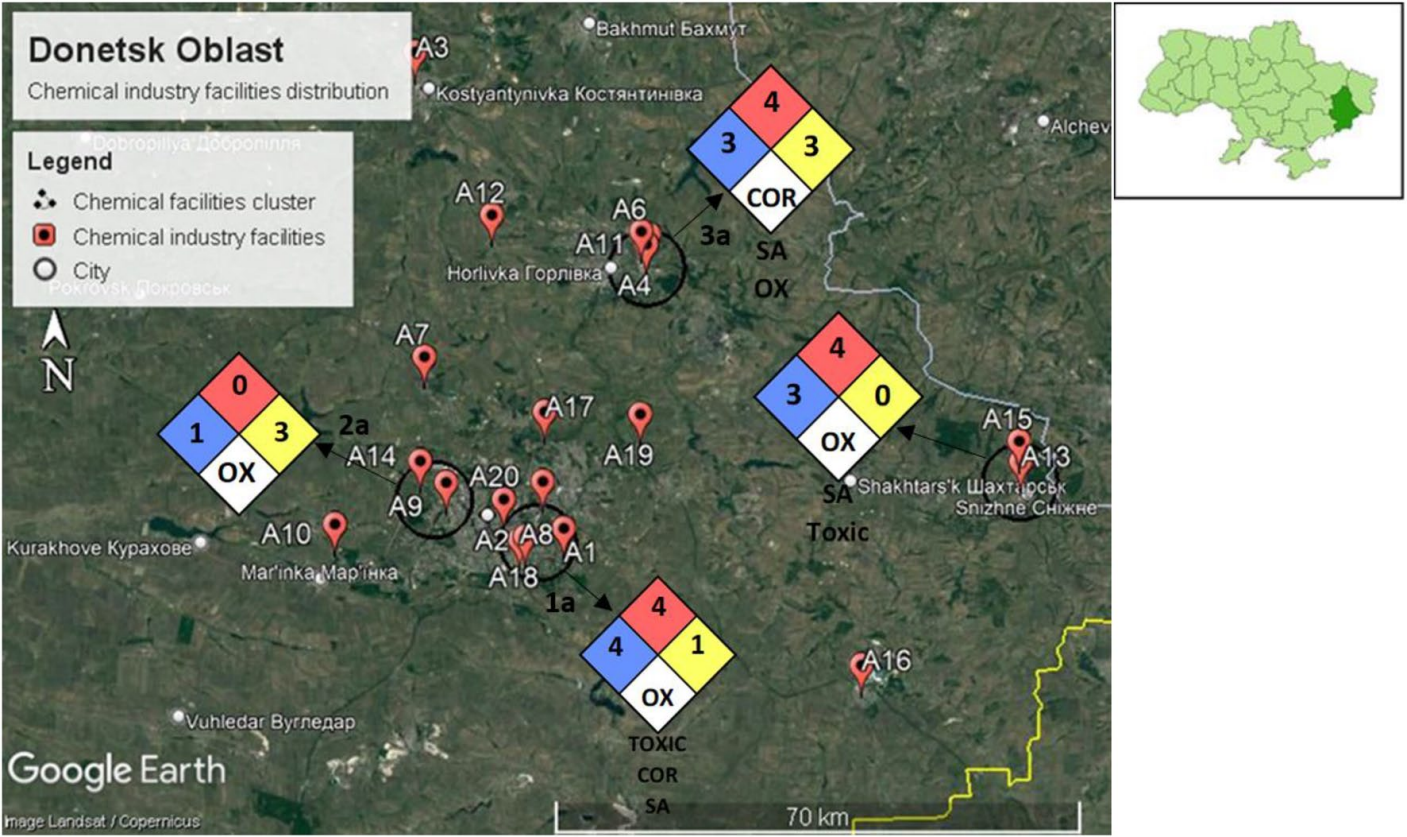
Chemical facilities cluster in Donetsk Oblast with NFPA hazard diamond assigned to each cluster.

Our chemical industry disaster risk assessment identified four chemical facilities cluster in Donetsk Oblast. The detailed chemical facilities cluster analysis is described in Table 2. All four chemical facilities cluster are prone to bombardment. Figure 10 shows the NFPA hazard diamond assigned to each cluster from the chemical facilities cluster analysis.

**Table 2:** Chemical facilities cluster analysis table for Donetsk Oblast.

No	Chemical facilities cluster name	Location and number of populations	Probability of bombardment	Chemical factory name and type	The chemicals involved in manufacturing	GHS classification	Potential hazards	Final custer NFPA 704
1	1a	Located within: City Donetsk—901,645	High	**A1**: Soap, detergents, cleaning products	• Alkali • Acids • Sodium hypochlorite	• Oxidizer • Irritant • Corrosive • Toxic • Environmental hazard	• Health Hazard: 4 • Flammability Hazard: 1 • Instability Hazard: 1 • Special Hazard: Corrosive	
				**A2**: Inorganic chemicals	• Inorganic acids • Sulfuric acid • Nitric acid	• Oxidizer • Toxic • Corrosive • Irritant • Environmental hazard	• Health Hazard: 4 • Flammability Hazard: 1 • Instability Hazard: 1 • Special Hazard: Corrosive	
				**A8:** Registered chemical factory with an unknown manufactured product	• Unknown	• Unknown	• Unknown	
				**A18:** Industrial gas oxygen, hydrogen, nitrogen, inert gases, propane etc	• Oxygen • Hydrogen • Nitrogen • Propane • Argon	• Oxidizer • Compressed gas • Flammable	• Health Hazard: 3 • Flammability Hazard: 4 • Instability Hazard: 0 • Special Hazard: Oxidizer, Simple asphyxiant	
				**A20**: Organic chemical, paint, and solvent	• Paint • Paint thinner	• Health hazard • Irritant • Flammable	• Health Hazard: 2 • Flammability Hazard: 3 • Instability Hazard: 0 • Special Hazard: Toxic	
2	2a	Located within: City Donetsk—901,645	High	**A9**: Registered chemical factory	• Unknown	• Unknown	• Unknown	
				**A14**: Industrial explosive and ammunition	• Dynamite • Ammonium Nitrate	• Explosive • Oxidizer • Irritant	• Health Hazard: 1 • Flammability Hazard: 0 • Instability Hazard: 3 • Special Hazard: Oxidizer	
3	3a	Located within: City Horlivka - 239,828	High	**A4**: Registered chemical factory with an unknown manufactured product	• Unknown	• Unknown	• Unknown	
				**A6**: Fertilizers, ammonia and urea	• Fertilizers • Ammonium nitrate • Ammonia • Urea • Phosphoric acid	• Irritant • Oxidizer • Compressed gas • Corrosive • Environmental hazard • health hazard	• Health Hazard: 3 • Flammability Hazard: 1 • Instability Hazard: 3 • Special Hazard: Simple asphyxiant, Corrosive	
				**A11**: Industrial gas oxygen, hydrogen, nitrogen, inert gases, propane etc	• Oxygen • Hydrogen • Nitrogen • Propane • Argon	• Oxidizer • Compressed gas • Flammable	• Health Hazard: 3 • Flammability Hazard: 4 • Instability Hazard: 0 • Special Hazard: Oxidizer, Simple asphyxiant	
4	4a	Located within: City Snizhne—45,767	High	**A13**: Industrial gas oxygen, hydrogen, nitrogen, inert gases, propane etc	• Oxygen • Hydrogen • Nitrogen • Propane • Argon	• Oxidizer • Compressed gas • Flammable	• Health Hazard: 3 • Flammability Hazard: 4 • Instability Hazard: 0 • Special Hazard: Oxidizer, simple asphyxiant	
				**A15**: Chemical machine manufacturing plant	• Petroleum crude oil • Silica • Polyvinyl chloride compounds	• Flammable • Irritant • Health hazard • Environmental hazard	• Health Hazard: 2 • Flammability Hazard: 4 • Instability Hazard: 0 • Special Hazard: Toxic

**Cluster 1a is** located southeast of the city of Donetsk, with an estimated population of 901,645. This cluster has chemicals with potentially lethal health hazards and flammability hazards that could vaporize and readily burn at ambient temperature. Furthermore, this cluster also contains instability hazards that could explode if preheated. Finally, cluster 1a has special hazards that potentially have oxidizing, toxic, corrosive, and simple asphyxiant.

**Cluster 2a** is located northwest of the city of Donetsk, with an estimated population of 901,645. This cluster has chemicals with health hazards that could cause significant irritation. However, this cluster does not have flammability hazards. Nevertheless, this cluster contains instability hazards that could explode at high temperatures or shock. Finally, cluster 2a has special hazards that are potential oxidizers.

**Cluster 3a** is located in the city of Horlivka, with an estimated population of 239,828. This cluster has chemicals with potential health hazards that could lead to serious or permanent injury and flammability hazards that could vaporize and readily burn at ambient temperature. In addition, this cluster contains instability hazards that could be ignited at ambient temperatures and pressures. Finally, cluster 3a has special hazards that are potentially corrosive, simple asphyxiant, and oxidizing.

**Cluster 4a** is located in the city of Snizhne, with an estimated population of 45,767. This cluster has chemicals with potential health hazards that could lead to serious or permanent injury and flammability hazards that could vaporize and readily burn at ambient temperature. However, it contains no instability hazards. Finally, cluster 3a has special hazards that are potentially oxidizing, simple asphyxiant, and toxic.

### Risk matrix for Donetsk Oblast

Due to ongoing war, the **likelihood** of chemical factories being bombarded in Donetsk Oblast is **High**.

**Figure 11:**
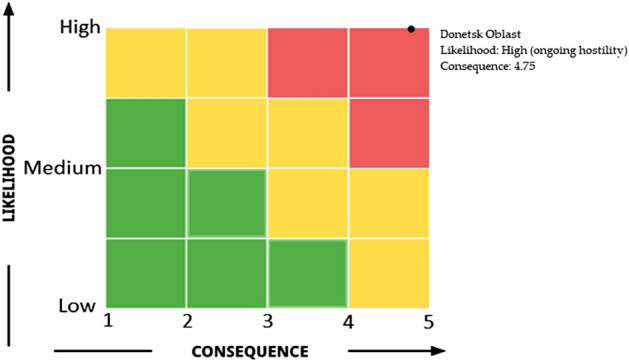
Disaster risk matrix for Donetsk Oblast.

We score the possible consequences of an attack on chemical facilities cluster to life and health, the environment, property, speed of development, and risk matrix score in Donetsk Oblast. The detail of the score assigned to each consequences indicator if the chemical facilities cluster is bombarded is shown in Table 3, and the risk matrix is illustrated in Fig. 11.

**Table 3:** Score assigned to each consequence indicator if the chemical facilities cluster is being bombarded.

Consequences of Life and Health	**5**. Catastrophic—Several deaths, (more than 20), hundreds of serious injuries, and more than 500 evacuated
Consequence for the environment	**5**. Catastrophic—Very heavy contamination, widespread effects
Consequences for property	**4**. Very serious—5–20 (total cost of damage Million USD)
Speed of development	**5**. No warning-immediate effect (explosion)
Average score	**4.75**

The disaster risk matrix for Donetsk Oblast shows a high likelihood of the chemical factory being bombarded due to the ongoing war. The average score of the consequences is 4.75.

### Luhansk Oblast

**Figure 12:**
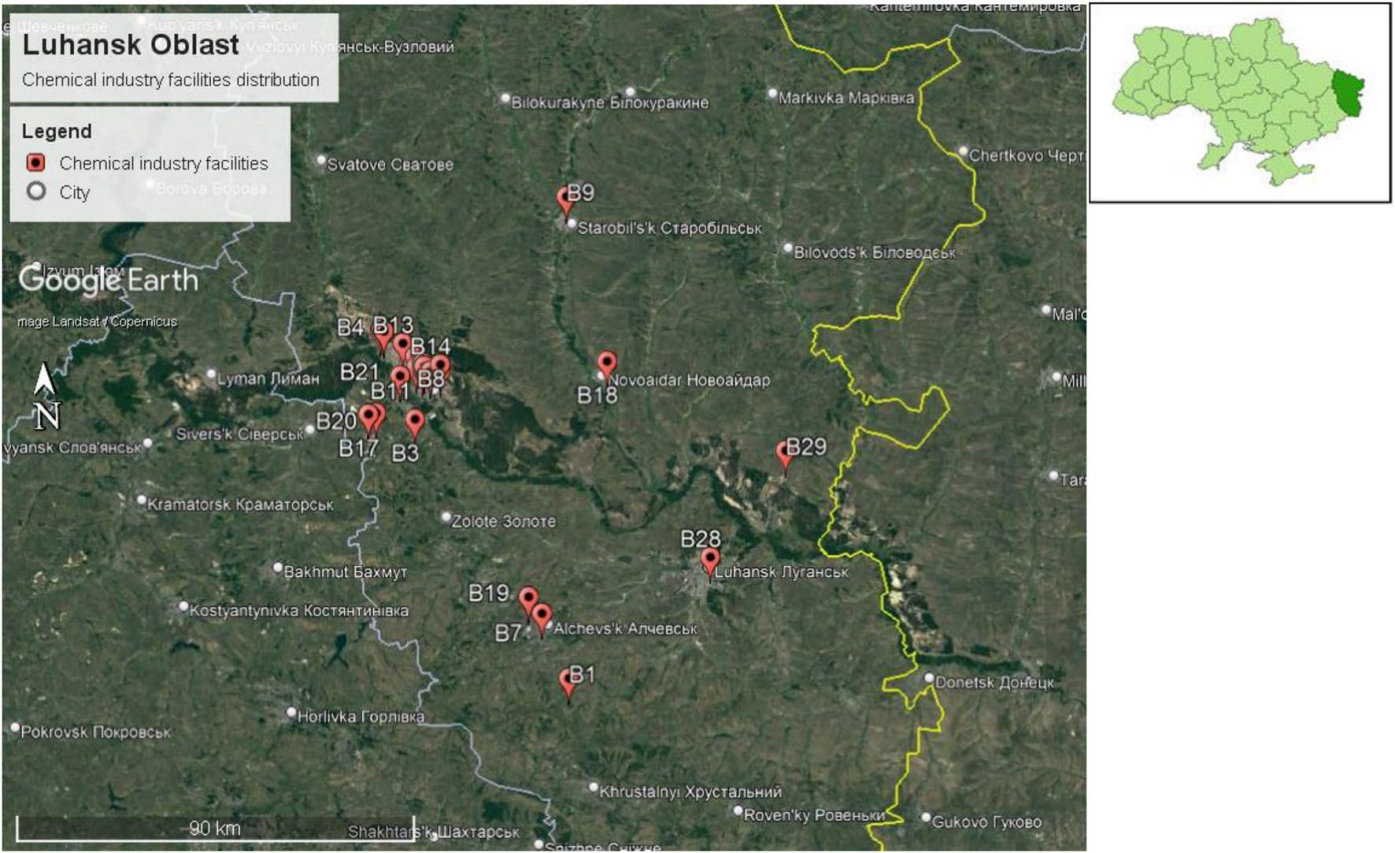
Chemical plant distribution in Luhansk Oblast in Ukraine.

The capital of Luhansk Oblast is Luhansk city, located between 48.5740° N, 39.3078° E. The size of Luhansk Oblast is 26,684 km^2^, with a population of 2,121,322, according to the 2021 estimation. Therefore, the population density of Luhansk Oblast is 79.5 persons/km^2^. Twenty-nine chemical factories were identified in Luhansk Oblast, as shown in Fig. 12.

**Figure 13:**
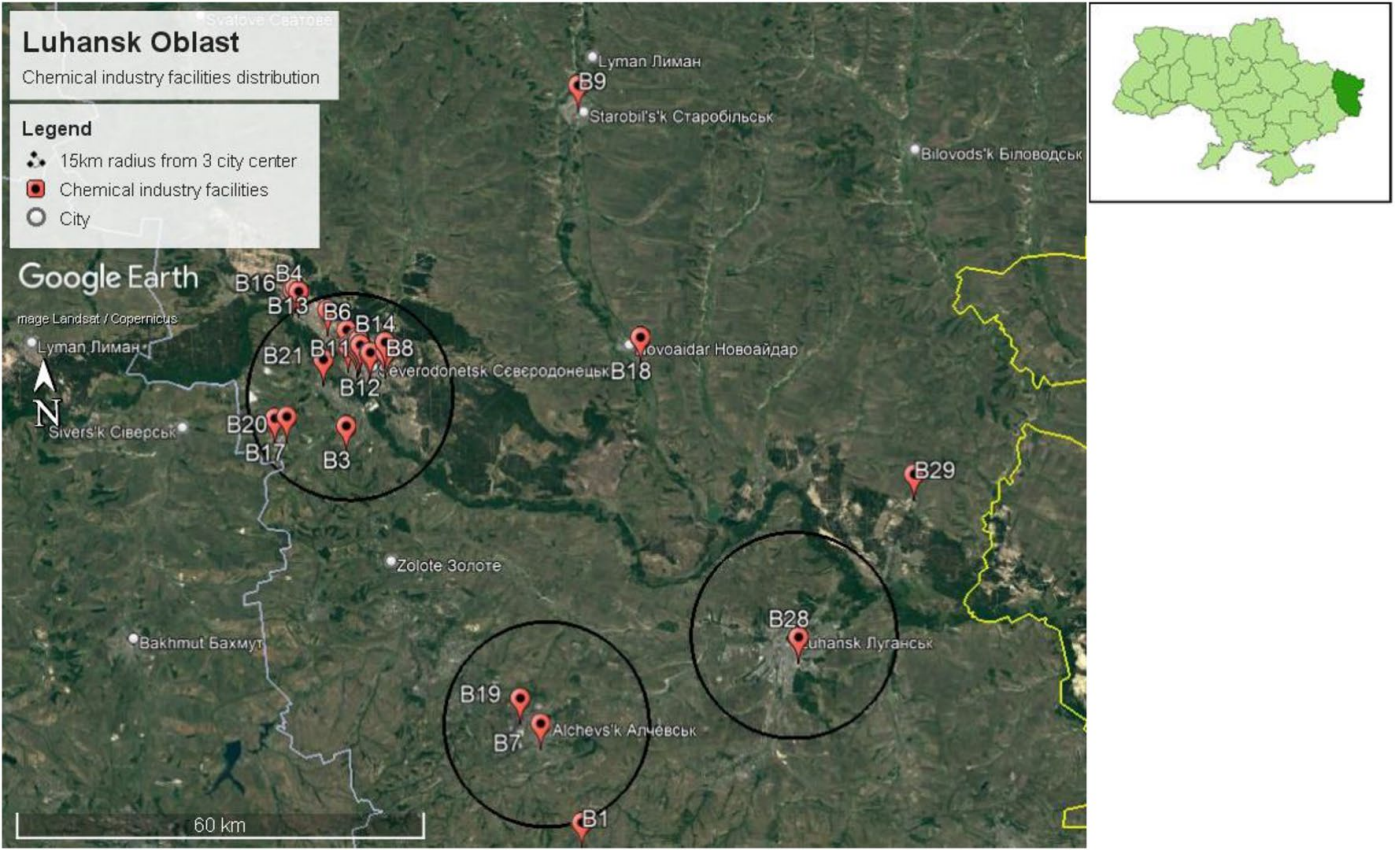
Chemical facilities identified within a 15 km radius of Luhansk city center, Alchevsk city center, and Lysychansk city center.

We have only one identified chemical facility within a 15 km radius of Luhansk city center. Nevertheless, we also identified two chemical facilities within a 15 km radius of Alchevsk city center. Furthermore, we identified 22 chemical facilities within a 15 km radius of Lysychansk city center, as shown in Fig. 13.

**Figure 14:**
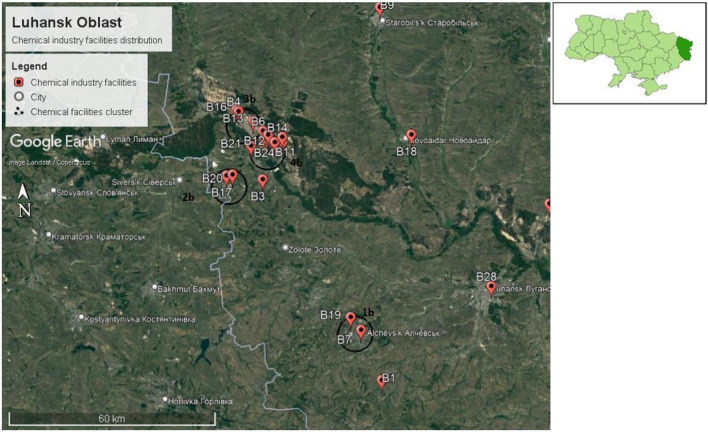
Chemical facilities cluster identified in Luhansk Oblast.

We identified four chemical plant clusters in Luhansk Oblast, namely, clusters 1b, 2b, 3b, and 4b, as shown in Fig. 14.

One cluster was identified in Alchevsk city (cluster 1b), 1 cluster in Verkhnokamyanka city(cluster 2b), 1 cluster in Rubizhne city(cluster 3b), and 1 cluster in Severodonetsk city(cluster 4b).

**Cluster 1b** is located in Alchevsk city, with an estimated population of 106,062.

Neighboring cities and settlements for cluster 1b are Karpaty, Mykhailivka, Malokostyantynivka, Perevalsk, Buhaivka, Horodnje, Seleznivka, Yashchykove, Artemivsk, Bryanka, and Kamyanka. These cities and settlements are located in the Alchevskyi district, with an estimated population of 93,340.

**Cluster 2b** is located in Verkhnokamyanka, a suburb of Lysynchansk city, with an estimated population of 93,340 .

**Cluster 1b** is located in Alchevsk city with a total estimated population of 106,062 persons.

Neighbouring cities and settlements for cluster 1b are Karpaty, Mykhailivka, Malokostyantynivka, Perevalsk, Buhaivka, Horodnje, Seleznivka, Yashchykove, Artemivsk, Bryanka, and Kamyanka. These cities and settlements are located in Alchevskyi district with a total estimated population of 93,340 persons.

**Cluster 2b** is located in Verkhnokamyanka, a suburb of Lysynchansk city with a total estimated population of 93,340 persons.

**Figure 15:**
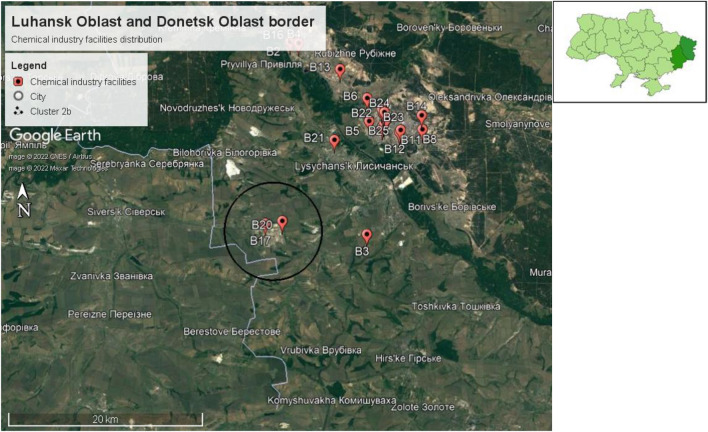
Chemical plant cluster 2b located near the border of Donetsk Oblast.

Neighboring cities and settlements for cluster 2b are Topolivka, Vovchoyarivka, Mykolaivka, Zolotarivka, Rai-Oleksandrivka, and Maloryazantseve. These cities and settlements are located in Sievierodonetskyi district, with an estimated population of 362,539 persons. Cluster 2b is also located on the border of Donetsk Oblast as shown in Fig. 15. Neighboring cities and settlements for cluster 2b in Donetsk Oblast are Verkhnokamyanske, Vyimka, Ivano-Darivka, Spirne, Siversk, and Serebrianka. These cities and settlements are located in the Bakhmutskyi district, with a total estimated population of 220,275 persons.

**Cluster 3b** is located in Rubizhne city, with an estimated population of 55,247.

Neighboring cities and settlements for cluster 3b are Vojevodivka, Severodonetsk, Synetskyi, Novodruzhesk, Shypylivka, Pryvillya, Stara Krasnyanka, Pshenychne, Varvarivka, and Kudryashivka. These cities and settlements are located in the Sievierodonetskyi district, with an estimated population of 362,539 persons.

**Cluster 4b** is located in Severodonetsk city, with an estimated population of 99,067.

Neighboring cities and settlements for cluster 3b are Vojevodivka, Severodonetsk, Synetskyi, Novodruzhesk, Shypylivka, Pryvillya, Stara Krasnyanka, Pshenychne, Varvarivka, and Kudryashivka. These cities and settlements are located in the Sievierodonetskyi district, with an estimated population of 362,539 persons.

**Figure 16:**
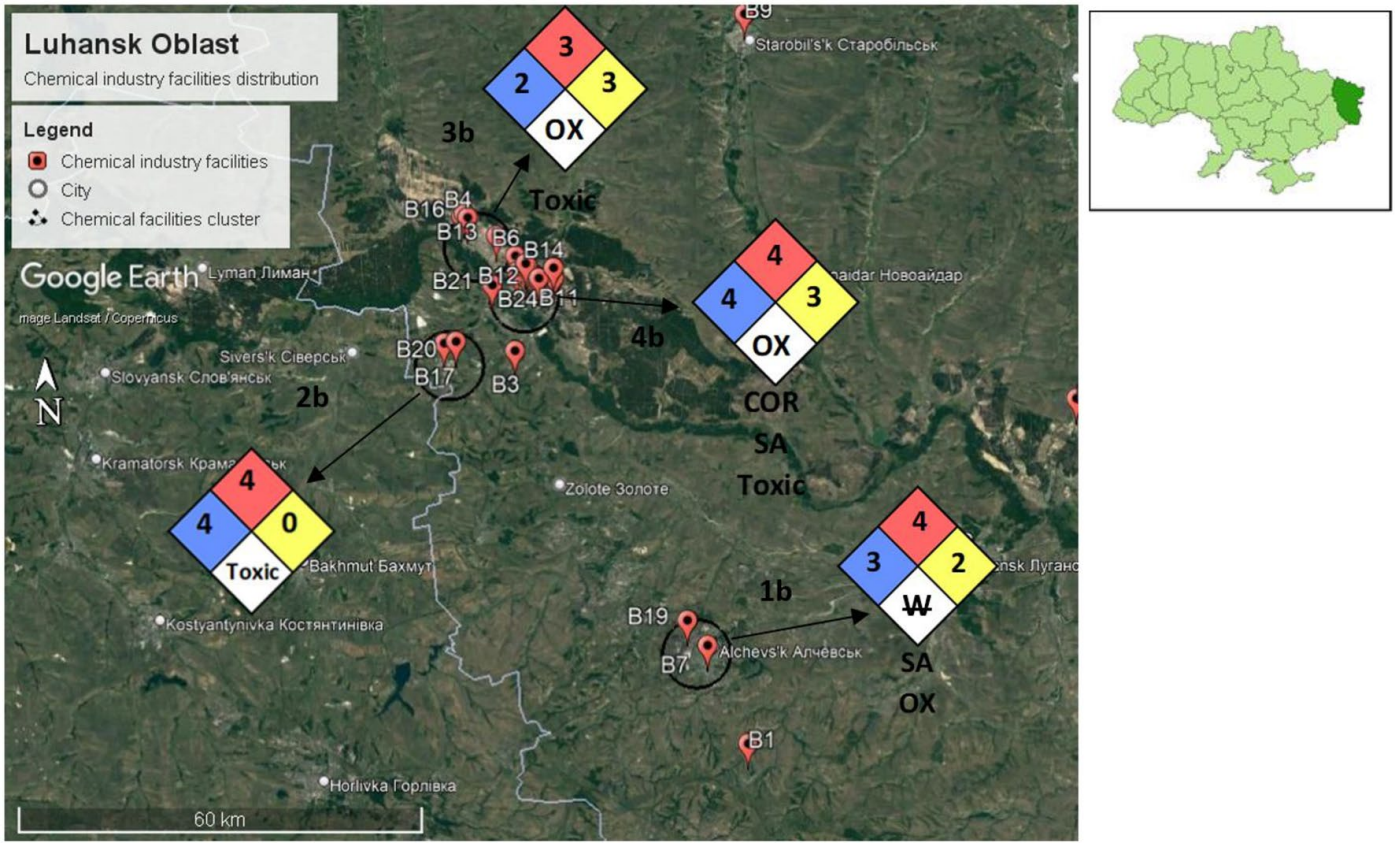
Chemical facilities cluster in Luhansk Oblast with an NFPA hazard diamond assigned to each cluster.

Our chemical industry disaster risk assessment identified four chemical facilities cluster in Luhansk Oblast. All four chemical facilities cluster are prone to bombardment. The detailed chemical facilities cluster analysis is described in Table 4. All four chemical facilities cluster are prone to bombardment. Figure 16 shows the NFPA hazard diamond assigned to each cluster from the chemical facilities cluster analysis.

**Table 4:** Cluster analysis table for Luhansk Oblast.

No	Chemical facilities cluster name	Nearby city and population (number of people)	Probability of bombardment	Chemical factory name and type	The chemicals involved in manufacturing	GHS classification	Potential hazards	Final cluster NFPA 704
1	1b	Located within: City Alchevsk—106,062	High	**B7**: Industrial gas, oxygen, hydrogen, nitrogen, inert gases, propane, etc	• Oxygen • Hydrogen • Nitrogen • Propane • Argon	• Oxidizer • Compressed gas • Flammable	• Health Hazard: 3 • Flammability Hazard: 4 • Instability Hazard: 0 • Special Hazard: Oxidizer, simple asphyxiant	
				**B19**: Coke and chemical factory	• Petroleum coke • Sulfuric acid	• Flammable • Corrosive • Toxic	• Health Hazard: 3 • Flammability Hazard: 1 • Instability Hazard: 2 Special Hazard: Reacts violently or explosively with water	
2	2b	Located within: Verkhnokamyanka, a suburban of Lysynchansk city—93,340	High	**B17**: Petrochemical	• Olefins • Benzene • Toluene • Hydrogen sulphide	• Flammable • Compressed gas • Irritant • Toxic • Health hazard • Environmental hazard	• Health Hazard: 4 • Flammability Hazard: 4 • Instability Hazard: 0 • Special Hazard: Toxic	
				**B20**: Petrochemical	• Olefins • Benzene • Toluene • Hydrogen sulphide	• Flammable • Compressed gas • Irritant • Toxic • Health hazard • Environmental hazard	• Health Hazard: 4 • Flammability Hazard: 4 • Instability Hazard: 0 • Special Hazard: Toxic	
3	3b	Located within: City Rubizhne—55,247	High	**B2**: Insecticide, fungicide, and herbicide	• Glyphosate • Boric acid • Malathion	• Irritant • Flammable • Health hazard • Environmental hazard	• Health Hazard: 2 • Flammability Hazard: 2 • Instability Hazard: 1 • Special Hazard: Toxic	
				**B4**: Organic chemical, paint, and solvent	• Paint • Paint thinner	• Health hazard • Irritant • Flammable	• Health Hazard: 2 • Flammability Hazard: 3 • Instability Hazard: 0 • Special Hazard: Toxic	
				**B13**: Industrial explosive and ammunition	• Dynamite • Ammonium Nitrate	• Explosive • Oxidizer • Irritant	• Health Hazard: 1 • Flammability Hazard: 0 • Instability Hazard: 3 • Special Hazard: Oxidizer	
				**B16**: Organic chemicals, polyurethane, and solvents	• Organic solvents • Polyurethane	• Irritant • Flammable • Health hazard • Environmental hazard	• Health Hazard: 2 • Flammability Hazard: 3 • Instability Hazard: 1 • Special Hazard: Toxic	
4	4b	Located within: City Sievierodonetsk—99,067	High	**B5**: Fertilizers, ammonia, and urea	• Fertilizers • Ammonium nitrate • Ammonia • Urea • Phosphoric acid	• Irritant • Oxidizer • Compressed gas • Corrosive • Environmental hazard • Health hazard	• Health Hazard: 3 • Flammability Hazard: 1 • Instability Hazard: 3 • Special Hazard: • Simple asphyxiant, Corrosive	
				**B6**: Wastewater treatment equipment and chemicals	• Sodium Aluminate • Chlorine • Calcium Oxide	• Irritant • Corrosive • Compressed gas • Toxic • Environmental hazard	• Health Hazard: 4 • Flammability Hazard: 0 • Instability Hazard: 1 • Special Hazard: Oxidizer, toxic	
				**B8**: Fertilizers, ammonia, and urea	• Fertilizers • Ammonium nitrate • Ammonia • Urea • Phosphoric acid	• Irritant • Oxidizer • Compressed gas Corrosive • Environmental hazard • Health hazard	• Health Hazard: 3 • Flammability Hazard: 1 • Instability Hazard: 3 • Special Hazard: Simple asphyxiant, Corrosive	
				**B10**: Industrial gas, oxygen, hydrogen, nitrogen, inert gases, propane,etc	• Oxygen • Hydrogen • Nitrogen • Propane • Argon	• Oxidizer • Compressed gas • Flammable	• Health Hazard: 3 • Flammability Hazard: 4 • Instability Hazard: 0 • Special Hazard: Oxidizer, simple asphyxiant	
				**B11**: Registered chemical factory with an unknown manufactured product	• Unknown	• Unknown	• Unknown	
				**B12**: Gas instrument manufacturer	• Petroleum crude oil • Silica • Polyvinyl chloride compound	• Flammable • Irritant • Health hazard • Environmental hazard	• Health Hazard: 2 • Flammability Hazard: 4 • Instability Hazard: 0 • Special Hazard: Toxic	
				**B14**: Construction materials and chemical	• Concrete and cement • Concrete curing compounds	• Irritant • Corrosive • Health hazard	• Health Hazard: 1 • Flammability Hazard: 0 • Instability Hazard: 0 • Special Hazard: Corrosive	
				**B15**: Nitric acid	• Nitric acid	• Oxidizer • Corrosive • Toxic	• Health Hazard: 4 • Flammability Hazard: 0 • Instability Hazard: 0 • Special Hazard: Oxidizing	
				**B21**: Petrochemical	• Olefins • Benzene • Toulene • Hydrogen sulphide	• Flammable • Compressed gas Irritant • Toxic • Health hazard • Environmental hazard	• Health Hazard: 4 • Flammability Hazard: 4 • Instability Hazard: 0 • Special Hazard: Toxic	
				**B22**: Chemical machine manufacturing plant	• Petroleum crude oil • Silica • Polyvinyl chloride compound	• Flammable • Irritant • Health hazard • Environmental hazard	• Health Hazard: 2 • Flammability Hazard: 4 • Instability Hazard: 0 • Special Hazard: Toxic	
				**B23**: Registered chemical factory with an unknown manufactured product	• Unknown	• Unknown	• Unknown	
				**B24**: Chemical machine manufacturing plant	• Petroleum crude oil • Silica • Polyvinyl chloride compound	• Flammable Irritant • Health hazard • Environmental hazard	• Health Hazard: 2 • Flammability Hazard: 4 • Instability Hazard: 0 • Special Hazard: Toxic	
				**B25**: Fertilizers, ammonia, and urea	• Fertilizers • Ammonium nitrate • Ammonia • Urea • Phosphoric acid	• Irritant • Oxidizer • Compressed gas • Corrosive • Environmental hazard • Health hazard	• Health Hazard: 3 • Flammability Hazard: 1 • Instability Hazard: 3 • Special Hazard: Simple asphyxiant, Corrosive	
				**B26**: Fertilizers, ammonia, and urea	• Fertilizers • Ammonium nitrate • Ammonia • Urea • Phosphoric acid	• Irritant • Oxidizer • Compressed gas • Corrosive • Environmental hazard • Health hazard	• Health Hazard: 3 • Flammability Hazard: 1 • Instability Hazard: 3 • Special Hazard: Simple asphyxiant, Corrosive	
				**B27**: Fertilizers, ammonia, and urea	• Fertilizers • Ammonium nitrate • Ammonia • Urea • Phosphoric acid	• Irritant • Oxidizer • Compressed gas • Corrosive Environmental hazard • Health hazard	• Health Hazard: 3 • Flammability Hazard: 1 • Instability Hazard: 3 • Special Hazard: Simple asphyxiant, Corrosive

**Cluster 1b** is located in Alchevsk, with an estimated population of 106,062. This cluster has chemicals with health hazards that could cause serious or permanent injury and flammability hazards that could vaporize and readily burn at ambient temperature. Furthermore, cluster 1b contains instability hazards that could cause violent chemical changes at high temperatures or pressure. This cluster also has special hazards that could react explosively with water, a simple asphyxiant, and oxidizing.

**Cluster 2b** is located in Verkhnokamyanka, a suburb of Lysynchansk city, with an estimated population of 93,340. This cluster has chemicals with potentially lethal health hazards and flammability hazards that could vaporize and readily burn at ambient temperature. This cluster also has special hazards that are toxic. However, cluster 2b contains no instability hazards.

**Cluster 3b** is located in the city of Rubizhne, with an estimated population of 55,247. This cluster has chemicals with health hazards that could potentially cause temporary incapacitation or residual injury and flammability hazards that could ignite at ambient temperature. In addition, cluster 3b contains instability hazards that could explode at high temperatures and pressures. Finally, this cluster also has special hazards that are oxidizing and toxic.

**Cluster 4b** is located in Sievierodonetsk, with an estimated population of 99,067. This cluster has chemicals with potentially lethal health hazards and flammability hazards that could vaporize and readily burn at ambient temperature. It also contains instability hazards that could explode at high temperatures and pressures and special hazards that are potentially oxidizing, a simple asphyxiant, and toxic.

### Risk Matrix for Luhansk Oblast

Due to ongoing war, the **likelihood** of chemical factories being bombarded in Luhansk Oblast is **High**.

**Figure 17:**
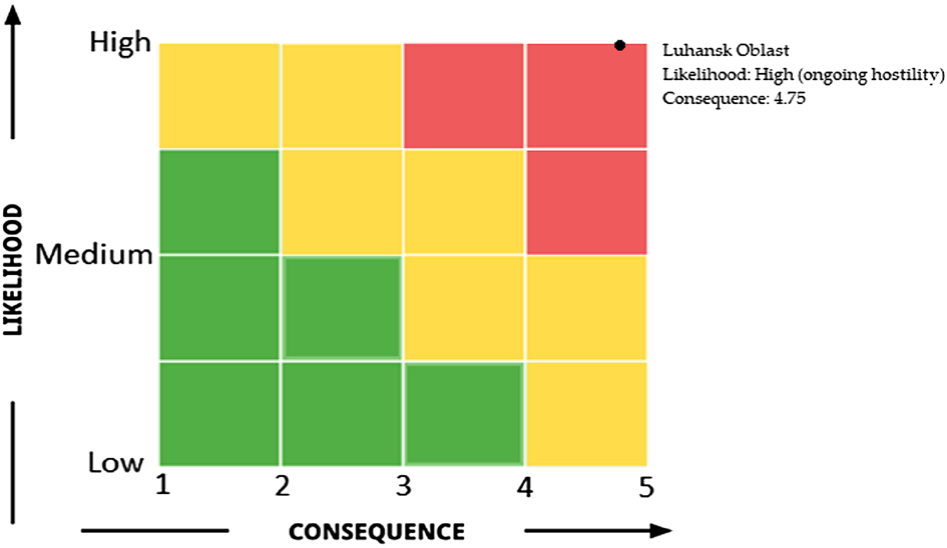
Disaster risk matrix for Luhansk Oblast.

We score the possible consequences of an attack on chemical facilities cluster to life and health, the environment, property, speed of development, and risk matrix score in Luhansk Oblast. The detail of the score assigned to each consequences indicator if the chemical facilities cluster is bombarded is shown in Table 5, and the risk matrix is illustrated in Fig. 17.

**Table 5:** Score assigned to each consequences indicator if the chemical facilities cluster being bombarded.

Consequences of Life and Health	**5**. Catastrophic—Several deaths, (more than 20), hundreds of serious injuries, more than 500 evacuated
Consequence for the environment	**5**. Catastrophic—Very heavy contamination, widespread effects
Consequences for property	**4**. Very serious—5–20 (total cost of damage Million USD)
Speed of development	**5**. No warning-immediate effect (explosion)
Average score	**4.75**

The disaster risk matrix for Luhansk Oblast shows a high likelihood of the chemical factory being bombarded due to the ongoing war. The average score of the consequences is 4.75.

## Summary

**Figure 18:**
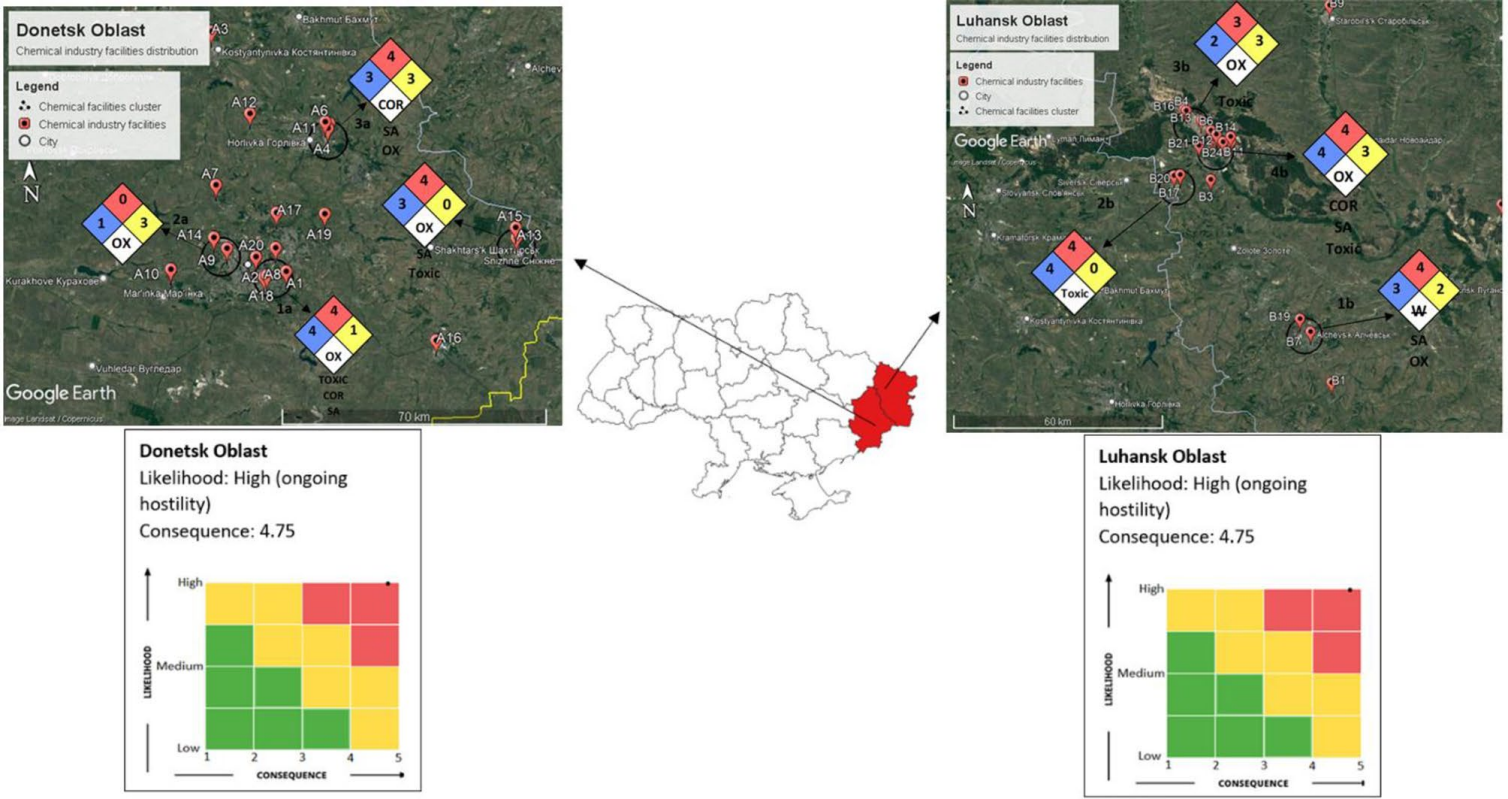
Chemical industry disaster risk assessment and risk metrics in Eastern Ukraine.

Our chemical industry disaster risk assessment on Donetsk Oblast shows that four chemical facilities clusters are exposed to indiscriminate bombardment. As a result, more than 1 million people (1,187,240 people) are exposed to potential hazards from the chemical facilities clusters, such as health hazards that could be lethal and flammability hazards. In addition, these chemical facilities clusters may contain instability hazards that could explode when attacked, and special hazards such as toxic, simple asphyxiant, corrosive, and reacting violently or explosively with water. Therefore, the disaster risk matrix scored high in terms of likelihood and consequences to life and health, environment, property, and speed of development, as shown in Fig. 18.

As for Luhansk Oblast, our chemical industry disaster risk assessment shows that four chemical facilities clusters are exposed to indiscriminate bombardment. As a result, more than 350 thousand people (353,716 people) are exposed to potential hazards from chemical facilities clusters, such as health hazards and flammability hazards that could vaporize and readily burn at ambient temperature. Furthermore, it contains instability hazards that could explode if bombarded, and special hazards such as toxic, simple asphyxiant, corrosive, and reacting violently or explosively with water. The disaster risk matrix scored high in terms of likelihood and consequences to life and health, environment, property, and speed of development, as shown in Fig. 18.

## Discussion

This chemical industry disaster risk assessment addresses the information gap on chemical industry disaster risk during the complex emergencies in Eastern Ukraine in Donetsk Oblast and Luhansk Oblast. Our risk assessment method can be applied to other Oblast in Ukraine. To date, no published risk analysis is available pertaining to this topic and its consequences to public health and the environment.

This risk assessment aligns with the United Nations Disaster Risk Reduction (UNDRR) Sendai Framework’s priorities^25^ on understanding disaster risk and enhancing disaster preparedness for effective response by enabling risk management prescription from three perspectives, (a) Local community level, (b) National level technical and operational, and (c) International strategy.

### Local community

As communities are typically the first to experience the direct impact of hazardous phenomena, it is crucial to prioritize community awareness with regards to potential chemical incidents. Specifically, community education initiatives should concentrate on recognizing the signs and symptoms of a chemical leak and how to prepare and respond in the event of such incidents. The Federal Emergency Management Agency (FEMA) offers various tools, such as 'An Introduction to Hazardous Materials,' which can be modified to train communities in Eastern Ukraine on recognizing terrorist use of toxic industrial chemicals as Weapons of Mass Destruction^26^. Additionally, this course can provide communities with knowledge on how to protect themselves during hazardous materials release incidents.

### National level (technical and operational)

Early warning systems for chemical leaks should be made available to all Ukrainians, especially those living in and around high-risk areas. An inclusive and accessible multi-hazard early warning systems report commissioned by the UNDRR includes useful recommendations such as building on existing connections and networks within communities, building and unlocking communities knowledge, facilitating community-based data collection and hazard monitoring, delivering an effective early-warning message, just to name a few^27^. These recommendations for a successful early warning system should be adapted into the context of Eastern Ukraine.

Hazmat equipment supply and training should be reinforced amongst the Ukrainian first responder to handle possible chemical incidents. Although hazmat personal protective equipment (PPE) is essential to protect individuals from chemical incidents, no equipment is appropriate for all individuals and threats. Therefore, PPE must be selected and used adequately according to the local context, hazards identified, and level of risk^28^.

The Initial Operation Response to chemical incidents and Specialist Operational Response decontamination protocol has proven effective in the United Kingdom^29^ and can be adapted to the context of Eastern Ukraine. Treatment facilities such as clinics and hospital emergency rooms should have a decontamination facility. Decontamination aims to eliminate substances from the skin, hair, mucosal surfaces, lungs, and gastrointestinal tract so that the chemical agent is neither absorbed by the patient nor transferred to the treating healthcare workers or first responder^30^.

The Ukranian Ministry of Environmental Protection has established a convenient dashboard that records war damages and environmental crimes, which includes the leakage of toxic substances from the chemical factory^31,32^. In addition, the Ukrainian government should keep track of hazardous chemical facilities and storage. Clear records and inventories will enable more accurate risk assessment, disaster preparedness, and response plan.

### International strategy

The International Humanitarian Law should be amended to include any attack on a chemical facility, which will give rise to a similar effect of using chemical weapons and other weapons of mass destruction, to be considered a war crime.

Thoughts should be placed in the recovery phase after the war to ensure more resilient and safer chemical industry facilities that stroke balance between indicators such as green economic growth, environmental protection, and social and welfare of the city that align with Sustainable Development Goals(SDGs) and Sendai Framework(SF)^33,34^. SDGs and SF outcomes are interconnected, and there are many synergies between these policy instruments^34^. Therefore, it should be used as the backbone of the post-war recovery in Eastern Ukraine.

For the long-term plan, landscape ecological risk assessment could be used to comprehensively evaluate the ecological environment condition and topographic and socioeconomic aspects of Eastern Ukraine from the effect of the war^35^. Furthermore, various new technology can be utilized to determine the consequence of war and the presence of pollutants, such as remote sensing technology, electrochemical techniques, magnetic sensors for monitoring hazardous pollutants in water resources, machine learning approaches and aerosol depth in air pollution prediction^36–39^.

## Limitation

The ongoing complex emergencies in Ukraine have made in-depth studies of chemical factories and their manufactured chemicals impossible for the time being. Moreover, the limited access to Eastern Ukraine due to the current war has made the identification of the exact chemical type, quality, and quantity, and their interaction with the environment very challenging. Therefore, this risk assessment does not take into consideration the dynamicity of chemical disaster risk with the influence of natural elements such as meteorological factor, water, season change, temperature, and wind direction.

Another limitation of this disaster risk assessment is limited data availability and lack of reliable information regarding the manufactured product of chemical factories. There are three registered chemical factories in Donetsk Oblast and one registered chemical factory in Luhansk Oblast with unknown manufactured products included in our cluster analysis. The assumption has to be made based on visualization of the factories by using Google Earth Pro to confirm the presence of industrial infrastructure. Including these registered chemical factories with unknown manufactured products enables us to determine the density of chemical industrial facilities in a cluster. Therefore, the higher the density of chemical industrial facilities in a cluster, the higher the likelihood of it being bombarded by the ongoing armed conflict, leading to compounded risk and more severe consequences.

## Conclusions

This chemical industry disaster risk assessment during complex emergencies in Eastern Ukraine found more than 1 million people (1,187,240 people) in Donetsk Oblast and more than 350 thousand people (353,716 people) in Luhansk Oblast are exposed to potential hazards from the chemical facilities clusters. Furthermore, the aggregation risk of bombardment of chemical facilities cluster in Eastern Ukraine is also high due to ongoing war. Therefore, the chemical industry disaster risks for Eastern Ukraine during complex emergencies in Donetsk Oblast and Luhansk Oblast are high in terms of likelihood and consequences to life and health, environment, property, and speed of development. Further detailed risk assessment on the type of chemical and their hazards should be conducted once the situation permits.

## Supplementary Information

Supplementary Information.

## Data Availability

The datasets used and/or analysed during the current study available from the corresponding author on reasonable request.

## References

[1] Ukraine Conflict | ACAPS [Internet]. [cited 2022 May 3]. https://www.acaps.org/country/ukraine/crisis/conflict.

[2] Ukraine | OCHA [Internet]. [cited 2022 May 3]. https://www.unocha.org/ukraine.

[3] Chapter 3: Risk | GAR [Internet]. [cited 2022 May 1]. https://gar.undrr.org/chapters/chapter-3-risk.

[4] Chemicals – UkraineInvest [Internet]. [cited 2022 May 2]. https://ukraineinvest.gov.ua/industries/chemicals/.

[5] Ukrainian National Waste Management Strategy | DLF attorneys-at-law [Internet]. [cited 2022 Apr 14]. https://dlf.ua/en/ukrainian-national-waste-management-strategy-until-2030-approved/#3.

[6] Organisation for the Prohibition of Chemical Weapons. Compendium of Correspondence shared by States Parties on Ukraine [Internet]. [cited 2022 Nov 9]. https://www.opcw.org/sites/default/files/documents/2022/CompendiumofcorrespondencesharedbyStatesPartiesonUkraine.pdf.

[7] Technological hazards and health risks in Ukraine [Internet]. [cited 2022 May 2]. https://www.who.int/emergencies/situations/ukraine-emergency/technological-hazards-and-health-risks-in-ukraine.

[8] Chemical Weapon | History, Facts, Types, & Effects | Britannica [Internet]. [cited 2022 May 2]. https://www.britannica.com/technology/chemical-weapon.

[9] Emergency in Ukraine: External Situation Report #6, published 7 April 2022 | ReliefWeb Mobile [Internet]. [cited 2022 May 2]. https://m.reliefweb.int/report/3835778/ukraine/emergency-ukraine-external-situation-report-6-published-7-april-2022?lang=fr.

[10] World Health Organization Europe. Emergency in Ukraine: External Situation Report #6 [Internet]. 2022 [cited 2022 May 2]. https://www.who.int/publications/i/item/WHO-EURO-2022-5152-44915-64177.

[11] Ukraine, Russian-backed separatists trade accusations over acid tank explosion | Reuters [Internet]. [cited 2022 May 2]. https://www.reuters.com/world/europe/ukraine-russian-backed-separatists-trade-accusations-over-acid-tank-explosion-2022-04-05/.

[12] Ammonia leak reported at chemicals plant in Ukraine’s besieged Sumy | Reuters [Internet]. [cited 2022 May 2]. https://www.reuters.com/world/europe/ammonia-leak-reported-chemicals-plant-ukraines-besieged-sumy-2022-03-21/.

[13] Disaster risk | UNDRR [Internet]. [cited 2022 May 20]. https://www.undrr.org/terminology/disaster-risk.

[14] Disaster Risk | Understanding Disaster Risk [Internet]. [cited 2022 May 2]. https://www.preventionweb.net/understanding-disaster-risk/component-risk/disaster-risk.

[15] Crichton D. The Risk Triangle. Nat disaster Manag [Internet]. 1991 [cited 2022 May 20];102–103. https://www.ilankelman.org/crichton/1999risktriangle.pdf.

[16] Hazard | UNDRR [Internet]. [cited 2022 Feb 24]. https://www.undrr.org/terminology/hazard

[17] Vulnerability | UNDRR [Internet]. [cited 2022 Feb 24]. https://www.undrr.org/terminology/vulnerability.

[18] Education in emergencies in South Asia: reducing the risks facing vulnerable children - Afghanistan | ReliefWeb [Internet]. [cited 2022 May 20]. https://reliefweb.int/report/afghanistan/education-emergencies-south-asia-reducing-risks-facing-vulnerable-children.

[19] Exposure | UNDRR [Internet]. [cited 2022 May 20]. https://www.undrr.org/terminology/exposure.

[20] Industrial facility Definition: 112 Samples | Law Insider [Internet]. [cited 2022 Jun 2]. https://www.lawinsider.com/dictionary/industrial-facility.

[21] United Nation. Globally Harmonized System of Classification and Labelling of Chemicals (GHS). 2021 [cited 2022 May 2]; https://unece.org/transport/standards/transport/dangerous-goods/ghs-rev9-2021.

[22] NFPA 704: Standard System for the Identification of the Hazards of Materials for Emergency Response [Internet]. [cited 2022 May 2]. https://www.nfpa.org/codes-and-standards/all-codes-and-standards/list-of-codes-and-standards/detail?code=704.

[23] State Statistics Service of Ukraine. Statistical Yearbook of Ukraine 2022. [cited 2022 Jun 2]; www.iaastat.kiev.ua.

[24] United Nations Environment Programme. Hazard Identification and Evaluation in a Local Community - Technical Report Series No. 12 [Internet]. https://wedocs.unep.org/20.500.11822/9164.

[25] Sendai Framework for Disaster Risk Reduction 2015–2030 | UNDRR [Internet]. [cited 2022 Jun 6]. https://www.undrr.org/publication/sendai-framework-disaster-risk-reduction-2015-2030.

[26] FEMA - Emergency Management Institute (EMI) Course | IS-5.A: An Introduction to Hazardous Materials [Internet]. [cited 2023 Jan 2]. https://training.fema.gov/is/courseoverview.aspx?code=is-5.a&lang=en.

[27] Inclusive and accessible multi-hazard early-warning systems | UNW WRD Knowledge Hub [Internet]. [cited 2023 Jan 2]. https://wrd.unwomen.org/explore/library/inclusive-and-accessible-multi-hazard-early-warning-systems.

[28] Hick JL, Thorne CD (2006). Personal protective equipment. Disaster Medicine.

[29] Collins S, Williams N, Southworth F, James T, Davidson L, Orchard E (2021). Evaluating the impact of decontamination interventions performed in sequence for mass casualty chemical incidents. Sci. Rep..

[30] Houston M, Hendrickson RG (2005). Decontamination. Crit. Care Clin..

[31] Eco Threat - Ministry of Environmental Protection Ukraine [Internet]. [cited 2023 Jan 2]. https://ecozagroza.gov.ua/.

[32] Ministry of Environmental Protection has calculated the cost of environmental damage caused by the war | Ukrayinska Pravda [Internet]. [cited 2023 Jan 2]. https://www.pravda.com.ua/eng/news/2022/06/23/7354265/.

[33] Karimian H, Qi L, Chen H (2013). Assessing urban sustainable development in Isfahan. Appl. Mech. Mater..

[34] SF and the SDGs | UNDRR [Internet]. [cited 2023 Jan 2]. https://www.undrr.org/implementing-sendai-framework/sf-and-sdgs.

[35] Karimian H, Zou W, Chen Y, Xia J, Wang Z (2022). Landscape ecological risk assessment and driving factor analysis in Dongjiang river watershed. Chemosphere.

[36] Karimian H, Li Y, Chen Y, Wang Z (2023). Evaluation of different machine learning approaches and aerosol optical depth in PM25 prediction. Environ. Res..

[37] Hojjati-Najafabadi A, Salmanpour S, Sen F, Asrami PN, Mahdavian M, Khalilzadeh MA (2022). A tramadol drug electrochemical sensor amplified by biosynthesized Au nanoparticle using mentha aquatic extract and ionic liquid. Top. Catal..

[38] Hojjati-Najafabadi A, Rahmanpour MS, Karimi F, Zabihi-Feyzaba H, Malekmohammad S, Agarwal S (2020). Determination of tert-butylhydroquinone using a nanostructured sensor based on CdO/SWCNTs and ionic liquid. Int. J. Electrochem. Sci..

[39] Hojjati-Najafabadi A, Mansoorianfar M, Liang T, Shahin K, Karimi-Maleh H (2022). A review on magnetic sensors for monitoring of hazardous pollutants in water resources. Sci. Total Environ..

