# Supplementary material for: Novel Google Maps and Google Earth application for chemical industry disaster risk assessment during complex emergencies in Eastern Ukraine
Source: Sci Rep. 2023 Apr 8;13:5758. doi: 10.1038/s41598-023-31848-6 (PMC10082827; doi:10.1038/s41598-023-31848-6)
Supplement: Supplementary file 1 — Supplementary Information. [file 41598_2023_31848_MOESM1_ESM.docx]

**Novel Google Maps and Google Earth Application for Chemical Industry Disaster Risk Assessment during Complex Emergencies in Eastern Ukraine**

Rick Kye Gan^1,2*^, Emanuele Bruni^1^, Rafael Castro Delgado^1,3^, Carlos Alsua^4^, and Pedro Arcos González^1^

^1^ Unit for Research in Emergency and Disaster, Public Health Area, Department of Medicine, University of Oviedo, Oviedo, Asturias, Spain

^2^ Department of Global Public Health, Karolinska Institutet, Stockholm, Sweden

^3^ Servicio de Salud del Principado de Asturias (SAMU-Asturias), Instituto de Investigación Sanitaria del Principado de Asturias, Oviedo, Asturians, Spain

^4^ McGuire Center for Entrepreneurship, University of Arizona, Tucson, USA

^*^ Correspondence: [rickkye91@gmail.com](mailto:rickkye91@gmail.com)

**ANNEX 1**

Owing to the limited information available regarding the type and amount of chemicals used as precursors and produced by the chemical factories in Eastern Ukraine, an assumption on the type of chemical hazard has to be made based on the type of activity and manufacturing production of the factories.

We identified the type of chemical factory via the factory's official website. This risk assessment assumed the type of chemical product and waste manufactured by the factory based on the type of chemical factory. The hazard property of these chemicals was determined by referring to the chemical data safety sheet. GHS classification (Globally Harmonized System of Classification and Potential Hazard) and NFPA 704 (Standard System for the Identification of the Hazards of Materials for Emergency Response) was constructed based on the chemical data safety sheet.

Table A and B shows the analysis of chemical factories based on available data.

**Table A: Cluster analysis Donetsk Oblast**

| Number | Cluster | Type of chemical factory | Probable chemical produced (Assumption) | Probable Classification (GHS) | Potential hazards |
| --- | --- | --- | --- | --- | --- |
| 1 | 1a | A1: Soap, detergents, cleaning products | Alkali ^1^  Acids ^2^  Sodium hypochlorite^3^ | - Oxidizer - Irritant - Corrosive - Toxic - Environmental hazard | - Health Hazard: 4 - Flammability Hazard: 1 - Instability Hazard: 1 - Special Hazard: Corrosive |
|  |  | A2: Inorganic chemicals | Inorganic acid ^4^  Sulfuric acid ^5^  Nitric acid ^2^ | - Oxidizer - Toxic - Corrosive - Irritant - Environmental hazard | - Health Hazard: 4 - Flammability Hazard: 1 - Instability Hazard: 1 - Special Hazard: Corrosive |
|  |  | A8: Registered chemical factory | Unknown | - Unknown | - Unknown |
|  |  | A18: Industrial gas oxygen, nitrogen, inert gases, propane etc | Oxygen ^6^  Hydrogen ^7^  Nitrogen ^8^  Propane ^9^  Argon ^10^ | - Oxidizer - Compressed gas - Flammable | - Health Hazard: 3 - Flammability Hazard: 4 - Instability Hazard: 0 - Special Hazard: Oxidizer, simple asphyxiant |
|  |  | A20: Organic chemical, paint, and solvent | Paint^11^  Paint thinner^12^ | - Health hazard - Irritant - Flammable | - Health Hazard: 2 - Flammability Hazard: 3 - Instability Hazard: 0 - Special Hazard: Toxic |
| 2 | 2a | A9: Registered chemical factory | Unknown | - Unknown | - Unknown |
|  |  | A14: Industrial explosive and ammunition | Dynamite ^13^  Ammonium Nitrate^14^ | - Explosive - Oxidizer - Irritant | - Health Hazard: 1 - Flammability Hazard: 0 - Instability Hazard: 3 - Special Hazard: Oxidizer |
| 3 | 3a | A4: Registered chemical factory | Unknown | - Unknown | - Unknown |
|  |  | A6: Fertilizers, ammonia  and urea | Fertilizer^15^  Ammonium nitrate ^14^  Ammonia^16^  Urea^17^  Phosphoric acid^18^ | - Irritant - Oxidizer - Compressed gas - Corrosive - Environmental hazard - Health hazard | - Health Hazard: 3 - Flammability Hazard: 1 - Instability Hazard: 3 - Special Hazard: Simple asphyxiant, Corrosive |
|  |  | A11: Industrial gas oxygen, hydrogen, nitrogen, inert gases, propane etc | Oxygen ^6^  Hydrogen ^7^  Nitrogen ^8^  Propane ^9^  Argon ^10^ | - Oxidizer - Compressed gas - Flammable | - Health Hazard: 3 - Flammability Hazard: 4 - Instability Hazard: 0 - Special Hazard: Oxidizer, simple asphyxiant |
| 4 | 4a | A13: Industrial gas oxygen, hydrogen, nitrogen, inert gases, propane etc | Oxygen ^6^  Hydrogen ^7^  Nitrogen ^8^  Propane ^9^  Argon ^10^ | - Oxidizer - Compressed gas - Flammable | - Health Hazard: 3 - Flammability Hazard: 4 - Instability Hazard: 0 - Special Hazard: Oxidizer, simple asphyxiant |
|  |  | A15: Chemical machine manufacturing plant | Petroleum crude oil^19^  Silica^20^  Polyvinyl chloride compound^21^ | - Flammable - Irritant - Health hazard - Environmental hazard | - Health Hazard: 2 - Flammability Hazard: 4 - Instability Hazard: 0 - Special Hazard: Toxic |

**Table B: Cluster analysis Luhansk Oblast**

| Number | Cluster | Type of chemical factory | Probable chemical produced (Assumption) | Probable Classification (GHS) | Potential hazards |
| --- | --- | --- | --- | --- | --- |
| 1 | 1b | B7: Industrial gas oxygen, nitrogen, hydrogen, inert gases, propane etc | Oxygen ^6^  Hydrogen ^7^  Nitrogen ^8^  Propane ^9^  Argon ^10^ | - Oxidizer - Compressed gas - Flammable | - Health Hazard: 3 - Flammability Hazard: 4 - Instability Hazard: 0 - Special Hazard: Oxidizer, simple asphyxiant |
|  |  | B19: Coke and chemical factory | Petroleum coke^22^  Sulfuric acid^5^ | - Flammable - Corrosive - Toxic | - Health Hazard: 3 - Flammability Hazard: 1 - Instability Hazard: 2 - Special Hazard: ~~W~~ Reacts violently or explosively with water |
| 2 | 2b | B17: Petrochemical | Olefins^23^  Benzene^24^  Toluene^25^  Hydrogen sulphide^26^ | - Flammable - Compressed gas - Irritant - Toxic - Health hazard - Environmental hazard | - Health Hazard: 4 - Flammability Hazard: 4 - Instability Hazard: 0 - Special Hazard: Toxic |
|  |  | B20: Petrochemical | Olefins^23^  Benzene^24^  Toluene^25^  Hydrogen sulphide^26^ | - Flammable - Compressed gas - Irritant - Toxic - Health hazard - Environmental hazard | - Health Hazard: 4 - Flammability Hazard: 4 - Instability Hazard: 0 - Special Hazard: Toxic |
| 3 | 3b | B2: Insecticide, fungicide and herbicide | Glyphosate^27^  Boric acid^28^  Malathion^29^ | - Irritant - Flammable - Health hazard - Environmental hazard | - Health Hazard: 2 - Flammability Hazard: 2 - Instability Hazard: 1 - Special Hazard: Toxic |
|  |  | B4: Organic chemical, paint and solvent | Paint^11^  Paint thinner^30^ | - Health hazard - Irritant - Flammable | - Health Hazard: 2 - Flammability Hazard: 3 - Instability Hazard: 0 - Special Hazard: Toxic |
|  |  | B13: Industrial explosive and ammunition | Dynamite^31^  Ammonium Nitrate^14^ | - Explosive - Oxidizer - Irritant | - Health Hazard: 1 - Flammability Hazard: 0 - Instability Hazard: 3 - Special Hazard: Oxidizer |
|  |  | B16: Organic chemicals, polyurethane and solvents | Organic solvent^32^  Polyurethane^33^ | - Irritant - Flammable - Health hazard - Environmental hazard | - Health Hazard: 2 - Flammability Hazard: 3 - Instability Hazard: 1 - Special Hazard: Toxic |
| 4 | 4b | B5: Fertilizers, ammonia and urea | Fertilizer^15^  Ammonium nitrate ^14^  Ammonia^16^  Urea^17^  Phosphoric acid^18^ | - Irritant - Oxidizer - Compressed gas - Corrosive - Environmental hazard - Health hazard | - Health Hazard: 3 - Flammability Hazard: 1 - Instability Hazard: 3 - Special Hazard: Toxic inhalation, Corrosive |
|  |  | B6: Waste water treatment equipment and chemicals | Sodium Aluminate^34^  Chlorine^35^  Calcium Oxide^36^ | - Irritant - Corrosive - Compressed gas - Toxic - Environmental hazard | - Health Hazard: 4 - Flammability Hazard: 0 - Instability Hazard: 1 - Special Hazard: Oxidizer, toxic |
|  |  | B8: Fertilizers, ammonia, and urea | Fertilizer^15^  Ammonium nitrate ^14^  Ammonia^16^  Urea^17^  Phosphoric acid^18^ | - Irritant - Oxidizer - Compressed gas - Corrosive - Environmental hazard - Health hazard | - Health Hazard: 3 - Flammability Hazard: 1 - Instability Hazard: 3 - Special Hazard: Toxic inhalation, Corrosive |
|  |  | B10: Industrial gas oxygen, hydrogen, nitrogen, inert gases, propane etc | Oxygen ^6^  Hydrogen ^7^  Nitrogen ^8^  Propane ^9^  Argon ^10^ | - Oxidizer - Compressed gas - Flammable | - Health Hazard: 3 - Flammability Hazard: 4 - Instability Hazard: 0 - Special Hazard: Oxidizer, simple asphyxiant |
|  |  | B11: Registered chemical factory | Unknown | - Unknown | - Unknown |
|  |  | B12: Gas instrument manufacturer | Petroleum crude oil^19^  Silica^20^  Polyvinyl chloride compound^21^ | - Flammable - Irritant - Health hazard - Environmental hazard | - Health Hazard: 2 - Flammability Hazard: 4 - Instability Hazard: 0 - Special Hazard: Toxic |
|  |  | B14: Construction materials and chemical | Concrete and cement^37^  Concrete curing compound^38^ | - Irritant - Corrosive - Health hazard | - Health Hazard: 1 - Flammability Hazard: 0 - Instability Hazard: 0 - Special Hazard: Corrosive |
|  |  | B15: Nitric acid | Nitric acid ^39^ | - Oxidizer - Corrosive - Toxic | - Health Hazard: 4 - Flammability Hazard: 0 - Instability Hazard: 0 - Special Hazard: Oxidizing |
|  |  | B21: Petrochemical | Olefins^23^  Benzene^24^  Toulene^25^  Hydrogen sulphide^26^ | - Flammable - Compressed gas - Irritant - Toxic - Health hazard - Environmental hazard | - Health Hazard: 4 - Flammability Hazard: 4 - Instability Hazard: 0 - Special Hazard: Toxic |
|  |  | B22: Chemical machine manufacturing plant | Petroleum crude oil^19^  Silica^20^  Polyvinyl chloride compound^21^ | - Flammable - Irritant - Health hazard - Environmental hazard | - Health Hazard: 2 - Flammability Hazard: 4 - Instability Hazard: 0 - Special Hazard: Toxic |
|  |  | B23: Unknown | Unknown | - Unknown | - Unknown |
|  |  | B24: Chemical machine manufacturing plant | Petroleum crude oil^19^  Silica^20^  Polyvinyl chloride compound^21^ | - Flammable - Irritant - Health hazard - Environmental hazard | - Health Hazard: 2 - Flammability Hazard: 4 - Instability Hazard: 0 - Special Hazard: Toxic |
|  |  | B25: Fertilizers, ammonia and urea | Fertilizer^15^  Ammonium nitrate ^14^  Ammonia^16^  Urea^17^  Phosphoric acid^18^ | - Irritant - Oxidizer - Compressed gas - Corrosive - Environmental hazard - Health hazard | - Health Hazard: 3 - Flammability Hazard: 1 - Instability Hazard: 3 - Special Hazard: Toxic inhalation, Corrosive |
|  |  | B26: Fertilizers, ammonia and urea | Fertilizer^15^  Ammonium nitrate ^14^  Ammonia^16^  Urea^17^  Phosphoric acid^18^ | - Irritant - Oxidizer - Compressed gas - Corrosive - Environmental hazard - Health hazard | - Health Hazard: 3 - Flammability Hazard: 1 - Instability Hazard: 3 - Special Hazard: Toxic inhalation, Corrosive |
|  |  | B27: Fertilizers, ammonia and urea | Fertilizer^15^  Ammonium nitrate ^14^  Ammonia^16^  Urea^17^  Phosphoric acid^18^ | - Irritant - Oxidizer - Compressed gas - Corrosive - Environmental hazard - Health hazard | - Health Hazard: 3 - Flammability Hazard: 1 - Instability Hazard: 3 - Special Hazard: Toxic inhalation, Corrosive |

Reference

1. Alkali Safety Data Sheet. [cited 2022 Aug 7]; Available from: https://www.smartjan.com/components/com_virtuemart/MSDS/SSS78002-MSDS.pdf

2. Nitric acid Safety Data Sheet [Internet]. 2021 [cited 2022 Aug 7]. Available from: https://www.fishersci.com/msds?productName=A467250%26productDescription=NITRIC

3. Sodium Hypochlorite Safety Data Sheet. [cited 2022 Aug 7]; Available from: https://www.sigmaaldrich.com/US/en/sds/sigald/425044

4. Inorganic Acid Core Flux Type 1 Safety Data Sheet. [cited 2022 Aug 7]; Available from: https://superiorflux.com/sds/Type_1_Inorganic_Acid_Core_Flux_Type_1.pdf

5. Sulfuric Acid Safety Data Sheet [Internet]. [cited 2022 Aug 7]. Available from: https://www.sigmaaldrich.com/US/en/sds/ALDRICH/339741

6. Oxygen Safety Data Sheet. [cited 2022 Aug 7]; Available from: https://www.airgas.com/msds/001043.pdf

7. SODIUM METASILICATE Safety Data Sheet: Composition/information on ingredients 3.1. Substance Substance type : Multi-constituent. [cited 2022 Nov 2]; Available from: https://www.gelest.com/wp-content/uploads/product_msds/SIS6982.0-msds.pdf

8. Nitrogen Safety Data Sheet [Internet]. [cited 2022 Aug 7]. Available from: https://www.airgas.com/msds/001040.pdf

9. Propane Safety Data Sheet. [cited 2022 Aug 7]; Available from: https://www.airgas.com/msds/001045.pdf

10. Argon Safety Data Sheet. [cited 2022 Aug 7]; Available from: https://www.airgas.com/msds/001004.pdf

11. Paint Safety Data Sheet. [cited 2022 Aug 7]; Available from: https://www.paintdocs.com/docs/webPDF.jsp?SITEID=PARA&prodno=9090&doctype=SDS&lang=2

12. Paint thinner safety data sheet [Internet]. [cited 2022 Nov 2]. Available from: https://monroetn.com/wp-content/uploads/2019/01/Paint-Thinner.pdf

13. Dynamite Safety Data Sheet [Internet]. [cited 2022 Nov 2]. Available from: https://www.austinpowder.com/wp-content/uploads/2021/08/SDS-P-11-Dynamite-AP.pdf

14. Ammonium Nitrate Safety Data Sheet [Internet]. [cited 2022 Aug 7]. Available from: https://www.chemicalstore.com/MSDS/SDS_Ammonium-Nitrate.pdf

15. Fertilizer Safety Data Sheet [Internet]. [cited 2022 Aug 7]. Available from: https://ictulsa.com/wp-content/uploads/2017/02/SDS-LB072107-05-30-15.pdf

16. Ammonia Safety Data Sheet. [cited 2022 Aug 7]; Available from: https://www.airgas.com/msds/001003.pdf

17. Urea Safety Data Sheet. [cited 2022 Aug 7]; Available from: https://www.sigmaaldrich.com/US/en/sds/sigma/u5378

18. Phosphoric Acid Safety Data Sheet [Internet]. [cited 2022 Aug 7]. Available from: https://beta-static.fishersci.com/content/dam/fishersci/en_US/documents/programs/education/regulatory-documents/sds/chemicals/chemicals-o/S25470B.pdf

19. Petroleum Crude oil Safety Data Sheet [Internet]. 2015 [cited 2022 Aug 7]. Available from: https://www.mercuria.com/sites/default/files/EN_SDS_Crude_SDS SGS GHS %28Reach ANNEXII%29_2015211_MERCURIA-37__0.pdf

20. Silica Safety Data Sheet [Internet]. [cited 2022 Aug 7]. Available from: https://web.faa.illinois.edu/app/uploads/sites/6/2021/05/Silica.pdf

21. Polyvinyl Chloride Compound (PVC) Safety Data Sheet. [cited 2022 Aug 7]; Available from: https://versatex.com/PDF/Versatex-SDS_16.pdf

22. Petroleum Coke Safety Data Sheet. [cited 2022 Aug 7]; Available from: https://www-s.nist.gov/m-srmors/msds/2719-MSDS.pdf

23. Olefins Safety Data Sheet [Internet]. [cited 2022 Aug 7]. Available from: https://www.marathonpetroleum.com/content/documents/Operations/Western_SDS/olefins.pdf

24. Benzene Safety Data Sheet [Internet]. [cited 2022 Aug 7]. Available from: https://www.airgas.com/msds/001062.pdf

25. Toulene Safety Data Sheet [Internet]. [cited 2022 Aug 7]. Available from: https://www.sigmaaldrich.com/US/en/sds/sial/244511

26. Hydrogen Sulphide Safety Data Sheet [Internet]. [cited 2022 Aug 7]. Available from: https://www.airgas.com/msds/001029.pdf

27. Glyphosate Safety Data Sheet [Internet]. [cited 2022 Aug 7]. Available from: https://alligare.com/wp-content/uploads/2019/06/glyphosate-5.4-sds-v3.1.pdf

28. Boric Acid Safety Data Sheet [Internet]. [cited 2022 Aug 7]. Available from: https://www.fishersci.com/msds?productName=AC315181000

29. Malathion Safety Data Sheet [Internet]. [cited 2022 Aug 7]. Available from: http://www.cdms.net/ldat/mp41D000.pdf

30. Paint Thinner Safety Data Sheet. [cited 2022 Aug 7]; Available from: https://westliberty.edu/health-and-safety/files/2013/08/Paint-thinner-kleanstrip.pdf

31. Dynamite Safety Data Sheet. [cited 2022 Aug 7]; Available from: http://sds.chemtel.net/webclients/ram/XQ266.pdf

32. Organic Solvent Safety Data Sheet [Internet]. [cited 2022 Aug 7]. Available from: https://rsc.aux.eng.ufl.edu/_files/msds/324.pdf

33. Polyurethane Coating Safety Data Sheet [Internet]. [cited 2022 Aug 7]. Available from: https://docs.rs-online.com/0b49/0900766b8113a600.pdf

34. Sodium Aluminate Safety Data Sheet [Internet]. 2020 [cited 2022 Aug 7]. Available from: https://www.alfa.com/en/msds/?language=EN&subformat=AGHS&sku=35453

35. Chlorine Safety Data Sheet [Internet]. [cited 2022 Aug 7]. Available from: https://www.airgas.com/msds/001015.pdf

36. Calcium Oxide Safety Data Sheet [Internet]. [cited 2022 Aug 7]. Available from: https://www.fishersci.com/store/msds?partNumber=AC196910010&productDescription=CALCIUM+OXIDE%2C+96%25+1KG&vendorId=VN00032119&countryCode=US&language=en

37. Safety Data Sheet Ready Mix Concrete [Internet]. [cited 2022 Aug 7]. Available from: https://www.lehighhanson.com/docs/default-source/safety-data-sheets/sds-ready-mix-concreteaa91c934fc6a4f02a7751e5afa0e0ea3.pdf?sfvrsn=412579e2_6

38. Concrete Curing Compound Safety Data Sheet [Internet]. [cited 2022 Aug 7]. Available from: https://www.wrmeadows.com/MSDS-PDF/3116250.pdf

39. Nitric acid Safety Data Sheet [Internet]. 2021 [cited 2022 Aug 7]. Available from: https://www.fishersci.com/msds?productName=A467250%26productDescription=NITRIC
